# PINK1 and PARKIN Deficiency Increases Neuronal Vulnerability to Autoimmune Neuroinflammation Through Mitochondrial Fragmentation in Aged Mice

**DOI:** 10.1111/jnc.70561

**Published:** 2026-10-23

**Authors:** Davide Cossu, Sachiko Noda, Shigeto Sato, Kazumasa Yokoyama, Yuji Tomizawa, Ruochen Wang, Sarah Vascellari, Leonardo Antonio Sechi, Taku Hatano, Nobutaka Hattori

**Affiliations:** ^1^ Department of Neurology Juntendo University Tokyo Japan; ^2^ Biomedical Research Core Facilities Juntendo University Tokyo Japan; ^3^ Department of Biomedical Sciences Sassari University Sassari Italy; ^4^ Neuron‐Glia Crosstalk Center Juntendo University Tokyo Japan; ^5^ Center for Biomedical Research Resources Juntendo University Tokyo Japan; ^6^ Department of Biomedical Sciences Cagliari University Cagliari Italy; ^7^ SC Microbiologia Azienda Ospedaliero Universitaria Sassari Italy; ^8^ Neurodegenerative Disorders Collaboration Laboratory RIKEN Center for Brain Science Wako Japan

**Keywords:** aging, EAE, mitochondrial fragmentation, mitophagy, neuroinflammation, PARKIN, PINK1, substantia nigra

## Abstract

PINK1 and Parkin regulate mitochondrial quality control in both neuronal and immune‐cell populations, but their roles during autoimmune neuroinflammation in aging remain incompletely understood. We investigated the association of PINK1 or Parkin deficiency with clinical disease, peripheral immune responses, CNS inflammation, and neuronal mitochondrial morphology during experimental autoimmune encephalomyelitis (EAE). EAE was induced in aged *PINK1* ^*−/−*^ and *PARK2* ^*−/−*^ mice and age‐matched controls. Clinical disease was assessed longitudinally, while independent cohorts were analyzed at peak disease using flow cytometry, spinal cord histology, transmission electron microscopy, and Western blotting of mitochondrial fractions. *PINK1* ^*−/−*^ and *PARK2* ^*−/−*^ showed greater cumulative disease burden and incomplete clinical recovery, most prominently in *PARK2* ^*−/−*^ mice. At peak disease, knockout mice exhibited increased dendritic cells, monocytes, and CD8^+^ T cells, while aged knockouts showed more IFN‐γ‐producing CD4^+^ and CD8^+^ T cells following ex vivo stimulation. Increased spinal cord inflammation was not accompanied by genotype‐dependent differences in myelin staining. Ultrastructural analysis revealed more numerous, smaller mitochondria in substantia nigra pars compacta and motor cortex neurons, accompanied by selective alterations in OPA1 and DRP1, but not MFN2. These findings demonstrate that PINK1 or Parkin deficiency is associated with enhanced inflammation, greater EAE disease burden, and altered neuronal mitochondrial morphology in aging. These results support an association between immune dysregulation and altered mitochondrial homeostasis without establishing impaired mitophagy or a direct causal relationship.

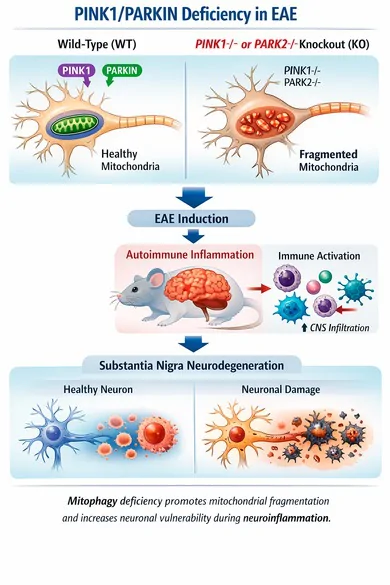

## Graphical abstract

PINK1 and Parkin help protect the nervous system during inflammation. When either protein is missing, autoimmune inflammation becomes more severe and neurons show greater mitochondrial abnormalities and damage, particularly with aging. These findings reveal how defective mitochondrial maintenance can make the brain more vulnerable to inflammatory injury.

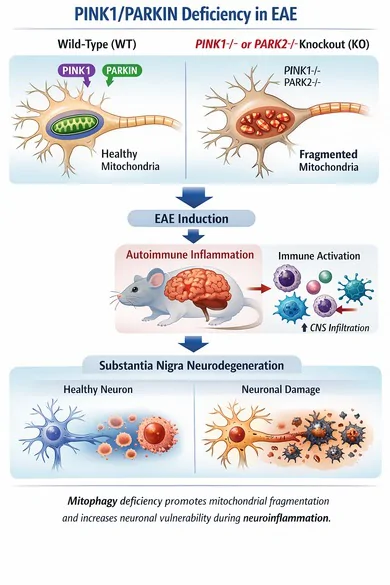

**Abbreviations**
ABC: avidin–biotin complex
ANOVA: analysis of variance
CFA: Complete Freund's adjuvant
ConA: Concanavalin A
EAE: experimental autoimmune encephalomyelitis
H&E: hematoxylin and eosin
IL: interleukin
LPS: lipopolysaccharide
MOG: Myelin oligodendrocyte glycoprotein
MS: multiple sclerosis
OVA: ovalbumin
PD: Parkinson's disease
PINK1: PTEN‐induced kinase 1
RRID: Research Resource Identifier (see scicrunch.org)
SD: standard deviation
SEM: standard error of the mean
TMEM119: transmembrane protein 119
TNFα: tumor necrosis factor alpha
WT: wild type

## 1 Introduction

Mitochondria are central regulators of both neuronal function and immune responses, providing not only energy but also key signaling platforms that govern cellular homeostasis. Mitophagy, the selective removal of damaged mitochondria, is critical to maintain mitochondrial quality control (Picca et al. 2023). With aging, mitophagy efficiency declines, leading to oxidative stress and chronic inflammation signaling (Jetto et al. 2022). These processes can compromise both neuronal survival and immune regulation, creating a vicious cycle that sustains neurodegeneration and neuroinflammatory processes (Faas and de Vos 2020; Marchi et al. 2023; Zhang et al. 2023).

The PTEN‐induced kinase 1 (PINK1, *PARK6*) and the E3 ubiquitin ligase PARKIN (*PARK2*) constitute a key pathway in mitochondrial quality control. Upon mitochondrial damage, PINK1 accumulates on the outer membrane and phosphorylates ubiquitin and PARKIN, initiating a cascade that labels dysfunctional mitochondria for autophagic removal (Hattori et al. 2014). Mutations in *PINK1* and *PARK2* cause autosomal recessive Parkinson's disease (PD), underscoring the importance of mitophagy in mitochondrial homeostasis (Quinn et al. 2020).

Beyond their canonical mitochondrial role, recent evidence indicates that PINK1 and PARKIN also modulate immune responses and neuroinflammation (Cossu et al. 2023; Quinn et al. 2020). In their absence, impaired mitochondrial quality control and mitophagy‐related pathways result in the accumulation of damaged mitochondria that sustain inflammatory signaling and alter immune cell activity (Quinn et al. 2020). Loss of these proteins enhances oxidative stress and promotes abnormal activation of microglia and macrophages, leading to increased production of pro‐inflammatory cytokines such as Tumor necrosis factor alpha (TNFα), interleukin (IL)‐1β, and IL‐6 (Mouton‐Liger et al. 2018). Furthermore, impaired mitophagy favors the presentation of mitochondrial antigens through MHC Class I molecules in macrophages and dendritic cells, thereby engaging adaptive immune mechanisms (Matheoud et al. 2016). In the brain, both proteins are upregulated in reactive astrocytes, where their deficiency exacerbates nitric oxide production, mitochondrial dysfunction, and glia‐mediated neurotoxicity (Sun et al. 2018). Collectively, PINK1 and PARKIN act as negative regulators of neuroinflammation by maintaining mitochondrial integrity and restraining excessive innate and adaptive immune activation.

Importantly, recent work has demonstrated that PINK1 also regulates peripheral myeloid cell activity following intestinal infections, promoting aberrant T cell activation and a pro‐inflammatory profile capable of inducing late onset motor dysfunction (Recinto et al. 2025). These findings highlight the capacity of mitochondrial quality control proteins to influence peripheral immunity, providing a mechanistic rationale for investigating neuroimmune interactions in aged *PARK2* ^*−/−*^ and *PINK1* ^*−/−*^ mice.

Experimental autoimmune encephalomyelitis (EAE) is a well‐established model of central nervous system autoimmunity and provides a controlled inflammatory framework to interrogate how mitochondrial quality control affects immune and neuroinflammatory responses. Although EAE is not a model of PD per se, it offers a useful system to dissect mitochondrial–immune interactions relevant to neurodegeneration. Previous studies in young *PARK2* ^*−/−*^ and *PINK1* ^*−/−*^ mice (8–10 weeks old) revealed aggravated inflammation and demyelination following EAE induction, suggesting that loss of mitophagy‐related genes enhances immune activation (Cossu et al. 2024; Cossu et al. 2022; Cossu, Yokoyama, Sato, et al. 2021). However, the contribution of aging, a major modifier of both mitochondrial function and immune competence, remains poorly understood.

In this study, we investigated neuroimmune responses in 18–20‐month‐old *PARK2* ^*−/−*^, *PINK1* ^*−/−*^, and wild‐type mice following active EAE induction. We aimed to determine how loss of *PINK1* or *PARKIN* affects immune cell dynamics and mitochondrial integrity under inflammatory stress. Dopaminergic neurons were analyzed as a high energy‐demanding and mitochondria‐dependent neuronal population, serving as a sensitive readout of mitochondrial vulnerability rather than a disease‐specific target of EAE pathology. Because PINK1 and PARKIN are strongly implicated in PD and mitochondrial homeostasis, substantia nigra neurons provide a particularly relevant neuronal population in which inflammatory stress may unmask latent mitochondrial defects. By integrating aging and inflammation, this work seeks to define mitochondrial–immune mechanisms that may contribute to both immune dysregulation and neuronal vulnerability across neuroinflammatory settings.

## 2 Materials and Methods

### 2.1 Ethics Statement

The study is reported in accordance with ARRIVE guidelines. All procedures involving animals were in compliance with the Ethics Guidelines for Animal Experimentation of Juntendo University School of Medicine and approved by the Institutional Animal Care and Use Committee of Juntendo University School of Medicine (ethics review approval No. 290238).

### 2.2 Mice and EAE Induction

Middle‐aged and aged female *PARK2* ^*−/−*^ and *PINK1* ^*−/−*^ mice were obtained from Charles River Laboratories Japan Inc. (Yokohama, Japan) at young age and subsequently aged and maintained in our animal facility until reaching the experimental time points, as previously described (Cossu et al. 2022; Cossu, Yokoyama, Sato, et al. 2021). Female mice were selected because EAE is generally more consistent and reproducible in female C57BL/6 mice, resulting in reduced inter‐animal variability and allowing a more reliable assessment of age‐ and genotype‐dependent effects while minimizing animal use. Age‐matched female littermates carrying no target mutation (*PARK2* ^*+/+*^ or *PINK1* ^*+/+*^), on the same C57BL/6 (H‐2b) genetic background, were used as wild type controls. Breeding was performed to maintain the colony; however, only age‐defined animals were included in the experimental procedures. Animals were housed in groups of 3–5 per cage according to genotype under specific pathogen‐free conditions at Juntendo University. Mice were identified by ear punching and grouped according to genotype, age, and sex. No formal randomization method was used to allocate animals to experimental groups. Instead, animals were assigned based on genotype and age, while ensuring balanced group sizes and consistent environmental conditions across cages to minimize potential confounding factors. Mice had ad libitum access to food and water and were maintained under a 12 h light/dark cycle (lights on at 07:00), with controlled temperature (22°C ± 2°C) and humidity (55% ± 10%).

Active EAE was induced by immunization with an emulsion of myelin oligodendrocyte glycoprotein (MOG)_35–55_ peptide (200 μg; sequence: MEVGWYRSPFSRVVHLYRNGK) obtained from Synpeptide Co. (Shanghai, China) in Complete Freund's adjuvant (CFA) supplemented with 4 mg/mL of *Mycobacterium tuberculosis* H37RA (Difco, BD Biosciences; Cat# 231141), followed by administration of pertussis toxin (200 ng/injections) (Difco, Detroit, MI, USA, Cat. No. 168‐22 471) on Day 0 and 48 h after. During immunization, mice were anesthetized with isoflurane (2%–3% in oxygen; Sigma‐Aldrich, Cat# 792632) delivered via inhalation using a calibrated vaporizer. Sham‐immunized controls received CFA and pertussis toxin at the same doses as the EAE‐immunized mice.

EAE severity was assessed using a clinical scoring scale ranging from 0 to 4. A score of 0 indicated no symptoms, while a score of 4 represented severe disability. Mild hind limb weakness or coordination deficits were scored as 1, whereas moderate symptoms, including partial hind limb paralysis and difficulty walking, were scored as 2. Severe hind limb paralysis, with the animal unable to walk or move properly, was classified as score 3. A score of 4 indicated complete paralysis of all four limbs, often resulting in immobility and necessitating euthanasia. To minimize pain and distress, humane endpoints were applied, including body weight loss exceeding 20%, a clinical score of ≥ 4.0, loss of the righting reflex at score 3, or refusal to eat and drink for two consecutive days. Mice reaching these criteria were euthanized under deep anesthesia using an overdose of pentobarbital (≥ 150 mg/kg, intraperitoneal injection; Sigma‐Aldrich, Cat# P3761), followed by cervical dislocation to confirm death.

Two independent cohorts were used for each genotype and age group. A longitudinal clinical cohort (*n* = 15 mice per group) was monitored until Day 39 post‐immunization for assessment of disease onset, progression, recovery, and cumulative disease scores. A separate peak‐disease cohort (*n* = 15 mice per group) was used for histological, ultrastructural, immunological, and ex vivo splenocyte analyses. An additional independent cohort of aged mice was used for Western blot analyses of mitochondrial proteins. For Western blot analyses, mice were euthanized under deep anesthesia with pentobarbital (≥ 150 mg/kg, intraperitoneal injection), followed by cervical dislocation to confirm death prior to tissue collection. All animals were monitored daily for clinical signs of EAE. The peak of clinical severity for each mouse was defined retrospectively as the highest clinical score reached during the disease course. All animals were monitored daily for clinical signs of EAE. In the longitudinal clinical cohort, the peak clinical score was defined retrospectively as the highest score recorded during the 39‐day observation period. For the tissue‐analysis cohort, a fixed day of euthanasia was not used because the timing of disease onset and peak severity varied according to genotype and age. Instead, tissue collection was based on the individual clinical disease stage. Animals were euthanized after reaching their maximal clinical score, once neurological deficits had stabilized or the first signs of clinical recovery were observed. Thus, animals were matched according to disease stage rather than chronological time after immunization. For histological, immunohistochemical, and ultrastructural analyses, mice were deeply anesthetized with pentobarbital (≥ 150 mg/kg, intraperitoneal injection) and transcardially perfused prior to tissue collection.

For immunological analyses, including T‐cell proliferation assays and flow cytometric profiling, mice were euthanized under deep anesthesia with pentobarbital (≥ 150 mg/kg, intraperitoneal injection), followed by cervical dislocation to confirm death prior to tissue harvesting.

A schematic overview of the experimental design, including group allocation, number of animals per group, and the stage‐based tissue collection strategy, is provided in Figure 1.

**FIGURE 1:**
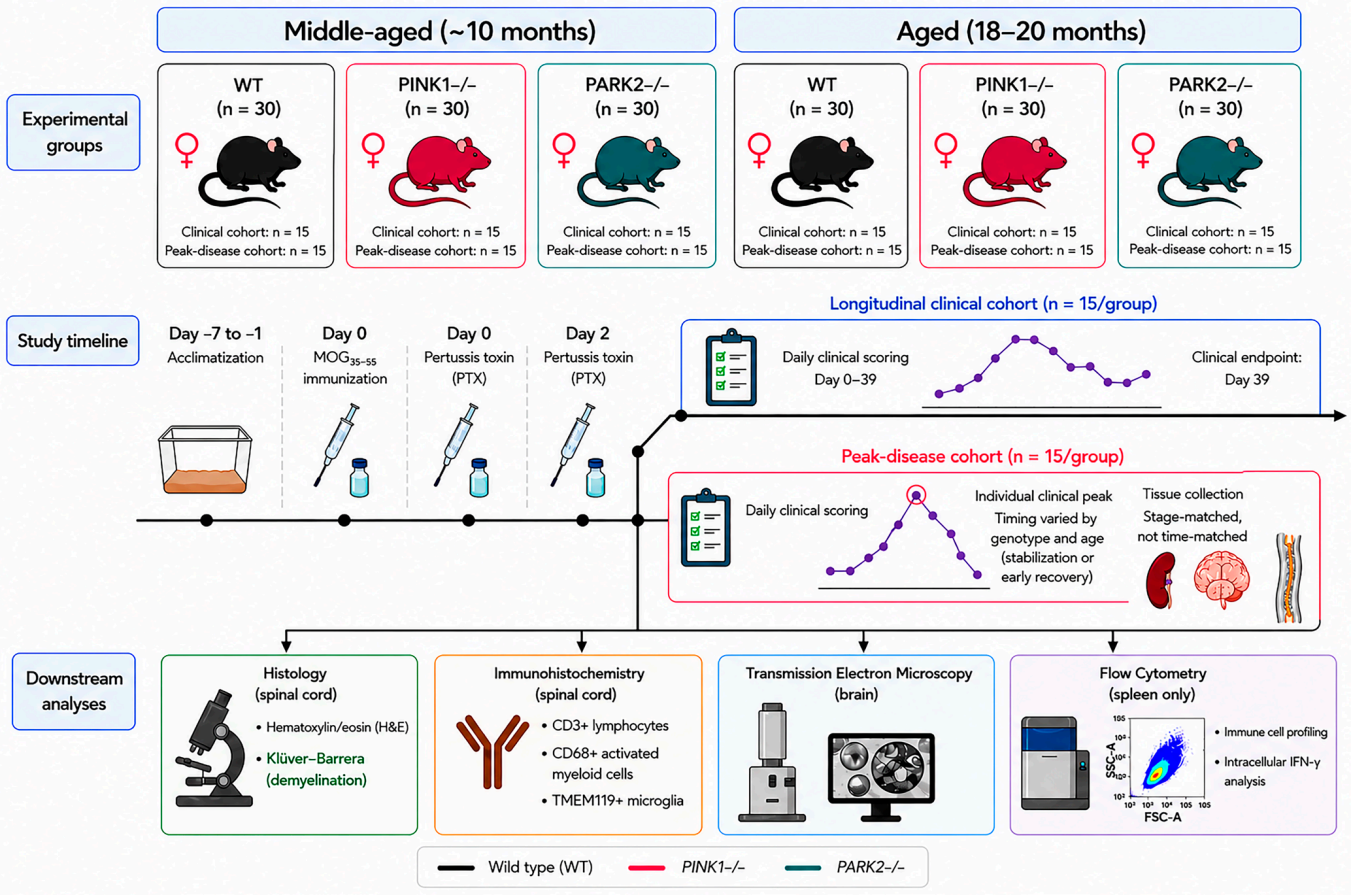
Experimental design and study timeline. Female wild type, *PINK1* ^*−/−*^ and *PARK2* ^*−/−*^ mice (total *n* = 30 per genotype and age group) were allocated to two independent cohorts: a longitudinal clinical cohort (*n* = 15 per group), monitored until Day 39 post‐immunization for assessment of disease onset, progression, recovery, and cumulative disease scores, and a peak‐disease cohort (*n* = 15 per group) used for histological, ultrastructural, immunological, and ex vivo splenocyte analyses. Mice were immunized with MOG_35–55_ peptide on Day 0, followed by pertussis toxin administration on Day 0 and 2. Clinical signs were monitored daily. Animals in the peak‐disease cohort were euthanized at maximal disease severity were euthanized at peak disease severity, defined as the maximal clinical score followed by stabilization or early recovery. Additional aged mice from an independent cohort were used for Western blot analyses of mitochondrial proteins. No animals were excluded from the study.

No a priori sample size calculation was performed. The number of animals per cohort (*n* = 15 per group) was determined based on previous studies employing similar EAE models and transgenic mouse lines, as well as on our prior experience, where comparable group sizes were sufficient to detect significant differences in clinical and immunological outcomes (Cossu et al. 2024; Cossu et al. 2022; Cossu, Yokoyama, Sato, et al. 2021).

### 2.3 Lymphocyte Stimulation Assays

To assess the impact of systemic *PARK2* and *PINK1* deletion on adaptive immune responses, spleen cell proliferation was measured after antigen‐specific and polyclonal stimulation. MOG_35–55_‐specific T‐cell responses were evaluated via [^3^H]‐thymidine incorporation (1 μCi/well; PerkinElmer, Waltham, MA, USA; Cat# NET027X), as previously described (Cossu et al. 2022). CD3^+^ T cells were isolated at the peak of EAE using the MojoSort Mouse CD3 Cell Isolation Kit (BioLegend; Cat# 480024) and cultured for 2 days with 50 μg/mL MOG_35–55_ peptide and gamma‐irradiated (3000 rad) syngeneic accessory spleen cells. Proliferation was assessed during the final 18 h using a microplate scintillation counter (MicroBeta TriLux, PerkinElmer).

To evaluate broader lymphocyte responsiveness, total splenocytes were also stimulated with lipopolysaccharide (LPS, 5 μg/mL; *Escherichia coli* O111:B4; Sigma‐Aldrich, St. Louis, MO, USA; Cat# L2630) or concanavalin A (ConA, 2 μg/mL; Sigma‐Aldrich; Cat# C5275). Cultures were performed under the same conditions, and proliferation was measured via [^3^H]‐thymidine incorporation. As negative controls, splenocytes from non‐immunized wild‐type mice were stimulated with an irrelevant peptide derived from ovalbumin (OVA_257_–_264_) (SIINFEKL; > 95% purity; Synpeptide, Shanghai, China).

### 2.4 Flow Cytometric Analysis

Single‐cell suspensions from spleens were prepared and stained for flow cytometry. Cells were first incubated with a live/dead discrimination dye (Zombie NIR Fixable Viability Kit, BioLegend; Cat# 423106) and pre‐blocked with anti‐mouse CD16/CD32 to reduce nonspecific binding, following standard protocols (Cossu et al. 2022). Surface staining was performed at 4 C for 30 min in the dark using fluorochrome‐conjugated monoclonal antibodies: CD3, CD4, CD8a, CD19, Ly6G, CD11b, CD115, CD11c, and I‐A/I‐E. Appropriate isotype‐matched controls were included for each staining panel.

For intracellular cytokine staining, splenocytes were stimulated ex vivo for 5 h at 37°C with a cell stimulation cocktail containing PMA and ionomycin in the presence of Brefeldin A (BioLegend Cell Activation Cocktail; Cat# 423303). Unstimulated samples (0 h) were processed in parallel and used as baseline controls. Cells were subsequently stained with a viability dye and surface antibodies against CD4 and CD8, followed by fixation and permeabilization using the Cyto‐Fast Fix/Perm Buffer Set (BioLegend) according to the manufacturer's instructions. Intracellular cytokine staining was then performed using fluorochrome‐conjugated antibodies against IFN‐γ. The frequencies of IFN‐γ‐producing CD4^+^ and CD8^+^ T cells were analyzed by flow cytometry.

Antibodies used in this study are listed in Table S1.

After staining, cells were washed and resuspended in PBS with 2% FBS for acquisition. A total of 20 000 live events per sample were acquired using a BD FACSCelesta flow cytometer (BD Biosciences, USA). Compensation was applied using single‐stained controls, and gating strategies were guided by fluorescence minus one control when appropriate. Data analysis was performed in FlowJo software (v10.10.0, FlowJo LLC), with gates set on singlets and on live lymphoid or myeloid populations as relevant. Total splenocyte counts and spleen weights were comparable across genotypes within the same age and treatment groups (data not shown), therefore, immune phenotyping was primarily reported as relative frequencies to highlight genotype‐ and age‐dependent changes in immune composition rather than differences driven by lymphoid expansion.

Dendritic cells and macrophages/monocytes were selected because they are key antigen‐presenting cells mediating MOG‐specific T‐cell priming in EAE, as supported by prior studies (Cossu et al. 2022; Cossu, Yokoyama, Sato, et al. 2021).

### 2.5 Histology and Immunohistochemistry of Brain and Spinal Cords

Brains and spinal cords were harvested from transcardially perfused mice, fixed in 10% neutral buffered formalin, and embedded for sectioning. Paraffin‐embedded tissue sections were stained with hematoxylin and eosin to evaluate the degree of inflammation. To assess myelin preservation, selected spinal cord sections were additionally stained using the Klüver–Barrera method, and the relative myelinated area was quantified using ImageJ thresholding analysis.

For immunohistochemistry, the Avidin‐Biotin Complex (ABC) kit (Vector Laboratories, Burlingame, CA, USA; Cat# PK‐6100) was used. Sections were incubated with primary antibodies including CD68, transmembrane protein 119 (TMEM119), and CD3. After primary antibody incubation, the sections were treated with biotinylated secondary antibodies, followed by the ABC reagent, which enhances the signal. Quantification of immune cell infiltration was performed in the white matter regions using Image J thresholding analysis, following intensity‐based segmentation to determine the area fraction of positive staining (Cossu et al. 2022; Cossu, Yokoyama, Sato, et al. 2021). This approach minimizes inter‐rater variability and bias. All analyses were performed in a blinded manner.

Inflammatory cells shown in Figure 4 were defined based on immunoreactivity for lineage‐specific markers (CD3 for T cells, CD68 for myeloid cells, and TMEM119 for microglia), with hematoxylin and eosin (H&E) staining providing morphological and anatomical context. Cellular morphology observed in H&E sections was consistent with the immunophenotypic identification.

### 2.6 Electron Microscopy

Mice were euthanized and transcardially perfused with 2.5% glutaraldehyde in 0.1 mol/L phosphate buffer (pH 7.2) for tissue fixation. Brain samples were post‐fixed, embedded in epoxy resin, and sectioned into ultrathin slices (70 nm) using an ultramicrotome. Sections were then mounted on grids and examined using a JEM‐1400 Flash electron microscope (JEOL, Japan). Mitochondria within dopaminergic neurons were manually quantified and measured for area using *cellSens* imaging analysis software (Olympus, Japan).

### 2.7 Mitochondrial Western Blot Analysis

Mitochondria were isolated from midbrain tissue using the Mitochondria Isolation Kit for Tissue (Cat. No. 89801, Thermo Fisher Scientific, Waltham, MA, USA) according to the manufacturer's instructions. All isolation buffers were supplemented immediately before use with Halt Protease and Phosphatase Inhibitor Cocktail, EDTA‐free (100×; Cat. No. 78441, Thermo Fisher Scientific). Isolated mitochondrial fractions were collected, and protein concentrations were determined using the Pierce BCA Protein Assay Kit (Cat. No. 23225, Thermo Fisher Scientific).

Equal amounts of mitochondrial protein (10 μg) were mixed with NuPAGE LDS Sample Buffer and Sample Reducing Agent (Thermo Fisher Scientific), denatured at 70°C for 10 min, and separated on NuPAGE 4%–12% Bis‐Tris Protein Gels using MES SDS Running Buffer (Thermo Fisher Scientific). Proteins were transferred onto PVDF membranes using the iBlot 3 Dry Blotting System with iBlot PVDF Transfer Stacks (Thermo Fisher Scientific), according to the manufacturer's protocol.

Membranes were blocked with StartingBlock T20 (TBS) Blocking Buffer (Thermo Fisher Scientific) for 1 h at room temperature and subsequently incubated overnight at 4°C with primary antibodies against OPA1, MFN2, or DRP1. COXIV was used as a mitochondrial loading control. Detailed information on the antibodies used, including manufacturer, catalog number, clone, RRID, and working dilution, is provided in Table S1. Following three washes in TBST, membranes were incubated with the appropriate HRP‐conjugated secondary antibodies for 2 h at room temperature.

Immunoreactive bands were visualized using SuperSignal West Pico PLUS Chemiluminescent Substrate (Thermo Fisher Scientific), and chemiluminescent signals were captured using the FUSION‐FX7. EDGE imaging system (Vilber Lourmat, Collégien, France). Band intensities were quantified using EVOLUTION‐Capt software (Vilber Lourmat).

OPA1 and MFN2 were assessed as proteins associated with mitochondrial fusion, whereas DRP1 was assessed as a protein associated with mitochondrial fission. For OPA1, long (L‐OPA1) and short (S‐OPA1) forms were quantified separately, and their signals were summed to determine total OPA1 abundance. The L‐OPA1/S‐OPA1 ratio was additionally calculated to assess the relative abundance of the two OPA1 forms. Total OPA1, MFN2, and DRP1 signals were normalized to COXIV from the corresponding sample and expressed as relative protein abundance.

### 2.8 Statistical Analysis

All statistical analyses were performed using GraphPad Prism v10.6.0 (GraphPad Software, La Jolla, CA, USA). Data distribution was assessed for normality using the Shapiro–Wilk test (Table S2). Based on these results, appropriate parametric or non‐parametric statistical tests were applied. Longitudinal data, including clinical score kinetics and body weight changes, were analyzed using two‐way repeated‐measures ANOVA, followed by Tukey post hoc test. This approach was applied despite deviations from normality, as it is commonly used for EAE clinical data and is considered robust to moderate violations of normality assumptions. Peak clinical scores, cumulative disease burden, and immunohistochemical data were analyzed using the Kruskal–Wallis test followed by Dunn's post hoc test, whereas quantification of the relative myelinated area following Klüver–Barrera staining was analyzed using one‐way ANOVA followed by Tukey's multiple comparisons test.

Western blot data for OPA1, DRP1, and MFN2 were analyzed separately for non‐EAE and EAE samples because samples from the two conditions were run on separate membranes. Within each membrane, differences among WT, *PINK1* ^*−/−*^, and *PARK2* ^*−/−*^ mice were analyzed using ordinary one‐way ANOVA followed by Dunnett's multiple‐comparisons test, with WT as the reference group. The L‐OPA1/S‐OPA1 ratio was analyzed using the same approach. No direct statistical comparisons were performed between non‐EAE and EAE samples.

Flow cytometry data, T‐cell proliferation, and electron microscopy data were analyzed using two‐way ANOVA followed by Tukey's post hoc test. Exact *p*‐values are reported in the corresponding figure legends. Full statistical reporting for all analyses is provided in Table S3. No formal outlier tests were performed, and all data points were included in the analyses.

## 3 Results

### 3.1 Differential Analysis of EAE Clinical Progression and Age‐Dependent T Cell Responses in Knockout Versus Wild‐Type Mice

All mice were monitored daily for weight changes and EAE clinical symptoms (Table 1). Figure 2 summarizes EAE progression across genotypes and age groups. Young mice profiles (A1–D1) extend previous observations (Cossu et al. 2022; Cossu, Yokoyama, Sato, et al. 2021), with new data from aged mice. Middle‐aged *PINK1* ^*−/−*^ mice exhibited earlier onset compared to wild type and *PARK2* ^*−/−*^ mice (*p* < 0.005), and both knockouts showed more severe symptoms than controls (*p* ≤ 0.001), with *PARK2* ^*−/−*^ mice reaching the highest peak scores (Figure 1C2). Recovery was observed in wild type but not in knockout mice. Aged *PINK1* ^*−/−*^ and *PARK2* ^*−/−*^ mice also showed earlier onset and increased severity (Figure 2A3–D3), with 40% of *PARK2* ^*−/−*^ mice developing chronic relapsing EAE. Longitudinal analysis confirmed significant effects of genotype, time, and their interaction across age groups (two‐way repeated measures ANOVA). Analysis of cumulative disease scores (Figure 2B1–B3; Kruskal–Wallis test) revealed a significant difference only in middle‐aged mice (*H* = 5.993, df = 2, *p* = 0.05), with post hoc Dunn's test indicating higher scores in *PINK1* ^*−/−*^ compared to wild‐type mice (*p* = 0.0433).

**TABLE 1:** Age‐and genotype‐related differences in the clinical course of EAE.

Longitudinal clinical cohort: *n* = 15 female mice per group	Active MOG_35–55_‐induced EAE					
Strain	Age at immunization	Incidence	Mortality	Day of disease onset	Maximum clinical score	CDS (Day 0–39)
Wild type	2 months	93%	0%	10 ± 1.3	2.4 ± 0.6	35.8 ± 0.7
*PINK1* ^*−/−*^		93%	6%	7 ± 1.0*	2.5 ± 0.5	45.3 ± 0.6*
*PARK2* ^*−/−*^		93%	0%	9 ± 2.0	3.3 ± 0.7*	50.4 ± 0.4*
Wild type	10 months	87%	6%	11 ± 0.5	2.6 ± 0.4	30.1 ± 0.8
*PINK1* ^*−/−*^		87%	6%	7 ± 1.0*	3 ± 0.5*	60.6 ± 0.7*
*PARK2* ^*−/−*^		87%	13%	12 ± 1.4	3.6 ± 0.5*	66.5 ± 0.9*
Wild type	18–20 months	87%	33%	15 ± 1.3	2.5 ± 0.4	34.2 ± 0.6
*PINK1* ^*−/−*^		73%	33%	11 ± 1.5*	2.7 ± 0.5	45.3 ± 0.8*
*PARK2* ^*−/−*^		87%	40%	12 ± 2.0*	3 ± 0.5*	55.5 ± 0.6*

*Note:* Data are presented as mean ± SEM. *p* < 0.05 versus age‐matched WT mice. Incidence and mortality are expressed as percentages. The * indicates statistical significance.

Abbreviation: CDS, cumulative disease score.

**FIGURE 2:**
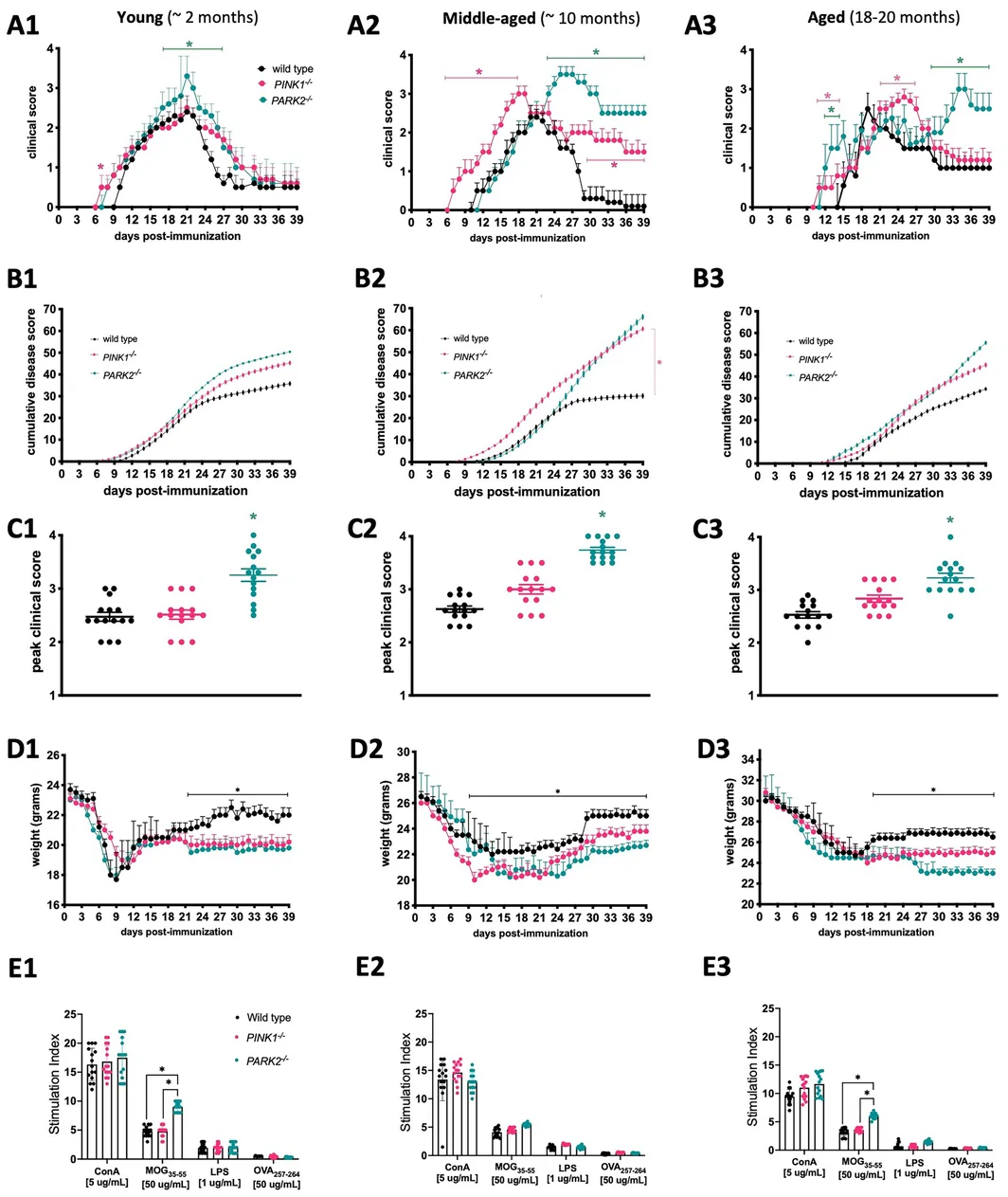
Comparative analysis of EAE disease progression in *PINK1* ^*−/−*^, *PARK2* ^*−/−*^ and wild type mice. Daily clinical scores (A1, A2, A3), cumulative disease scores (B1, B2, B3), peak clinical scores (C1, C2, C3), body weights (D1, D2, D3), and antigen‐specific stimulation of splenocytes by T‐cell proliferation (E1, E2, E3) are shown. Longitudinal data (clinical score kinetics and body weight) were analyzed using two‐way repeated‐measures ANOVA followed by Tukey's multiple comparisons test. T‐cell proliferation was analyzed using two‐way ANOVA followed by Tukey's multiple comparisons test. Peak clinical scores and cumulative disease scores were analyzed using the Kruskal–Wallis test followed by Dunn's multiple comparisons test. For cumulative disease scores, statistical analysis was performed on the final cumulative value for each individual animal. Data are presented as mean ± standard error of the mean (SEM). Each group included 15 mice. Data are pooled from three independent experiments (*n* = 5 mice per experiment; total *n* = 15 per group). *p* values versus wild type are indicated by asterisks.

Age‐dependent T cell responses were assessed by ConA and MOG_35–55_ stimulation (Figure 2E1–E3). In young mice, two‐way repeated measures ANOVA revealed a significant interaction between genotype and stimulation (*F*(2.317, 48.66) = 7.255, *p* = 0.0011). Post hoc Tukey's test showed increased proliferation in *PARK2* ^*−/−*^ mice compared to wild‐type controls under MOG_35–55_ (*p* < 0.0001), as well as compared to *PINK1* ^*−/−*^ mice (*p* < 0.0001).

In middle‐aged mice, no significant interaction between genotype and stimulation was observed (*F*(2.162, 45.40) = 5.049, *p* = 0.0607), indicating attenuation of genotype‐dependent differences.

In aged mice, a significant interaction between genotype and stimulation was detected (*F*(2.556, 53.6) = 7.965, *p* < 0.0001), with post hoc analysis revealing multiple significant differences across stimulation conditions, particularly involving *PARK2* ^*−/−*^ mice.

ConA elicited robust proliferation in all genotypes, with age‐related decline but no genotype differences. MOG‐specific responses displayed a biphasic pattern, with enhanced proliferation in young *PARK2* ^*−/−*^ mice, attenuation in middle‐aged animals, and re‐emergence in aged mice. In contrast, LPS induced weak proliferative responses across all groups, while OVA_257_–_264_ stimulation resulted in minimal proliferation, confirming antigen specificity. Non‐immunized control mice showed only baseline proliferation levels.

### 3.2 Flow Cytometric Characterization of Splenic Immune Responses at Peak Disease

Flow cytometric analysis of T‐cell populations in the spleens of EAE (MOG_35–55_‐immunized) mice at peak disease revealed a significant increase in CD8^+^ lymphocytes in both *PINK1* ^*−/−*^ and *PARK2* ^*−/−*^ mice compared to wild‐type controls across all age groups (Figure 3A1–A3). Two‐way ANOVA showed significant effects of genotype and age, as well as a genotype × age interaction (*p* < 0.0001 for genotype and age; *p* = 0.0001 for interaction). Post hoc Tukey's multiple comparisons test confirmed that both *PINK1* ^*−/−*^ and *PARK2* ^*−/−*^ mice exhibited higher percentages of CD8^+^ cells than age‐matched wild‐type mice in young, middle‐aged, and aged groups (all *p* < 0.0001), while no significant differences were observed between the two knockout genotypes. In contrast, no significant differences were detected in the proportions of CD4^+^ T cells between knockout and wild‐type mice.

**FIGURE 3:**
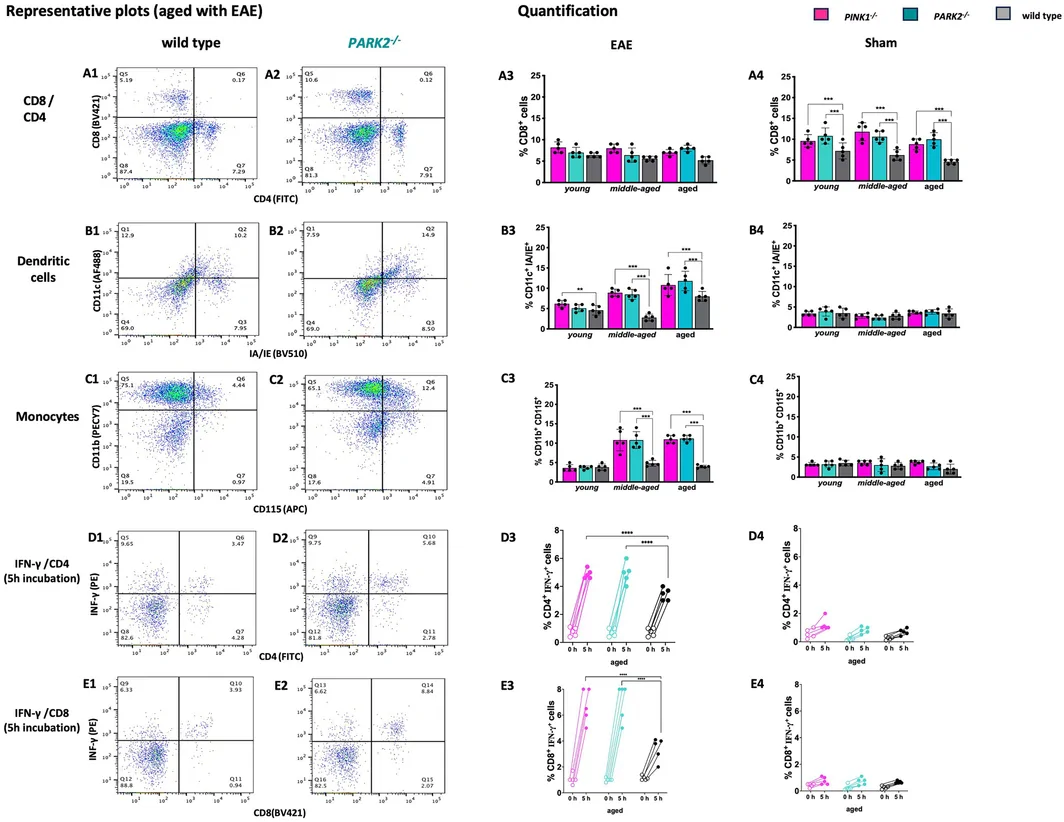
Enhanced splenic immune activation in *PINK1* ^*−/−*^ and *PARK2* ^*−/−*^ mice during peak EAE. Representative flow cytometry plots show CD4^+^ and CD8a^+^ T cells (A1, A2), CD11c^+^ IA/IE^+^ dendritic cells (B1, B2), CD11b^+^ CD115^+^ monocytes (C1, C2), intracellular IFN‐γ staining in CD4^+^ T cells (D1, D2), and intracellular IFN‐γ staining in CD8^+^ T cells (E1, E2). Corresponding quantitative analyses are shown for MOG_35–55_ immunized (A3, B3, C3, D3, E3) and sham‐immunized mice (A4‐E4). For intracellular cytokine staining, splenocytes were stimulated ex vivo with PMA and ionomycin for 5 h in the presence of brefeldin A. Representative plots from aged WT and *PARK2* ^*−/−*^ mice are shown; *PARK2* ^*−/−*^ mice were selected as representative because no significant differences were detected between the two knockout strains. Each dot represents one animal (*n* = 5 animals per group). Data are presented as mean ± SEM. Panels A–C were analyzed using two‐way ANOVA followed by Tukey's multiple‐comparisons test. For panels D–E, genotype comparisons were performed on the values obtained after 5 h of stimulation using one‐way ANOVA followed by Tukey's multiple‐comparisons test *p < 0.05; **p < 0.01; ***p < 0.001; ****p < 0.0001.

Flow cytometric analysis of dendritic cell populations revealed an increased frequency of conventional CD11c^+^ I‐A/I‐E^+^ dendritic cells in knockout mice during EAE (Figure 3B1–B3). Two‐way ANOVA showed significant effects of genotype and age, as well as a genotype × age interaction (*p* < 0.0001 for all factors). Post hoc Tukey's multiple comparisons test indicated that young *PINK1* ^*−/−*^ mice exhibited a higher percentage of dendritic cells compared to wild‐type controls (*p* = 0.0051), whereas no significant difference was observed in *PARK2* ^*−/−*^ mice at this age. In contrast, both *PINK1* ^*−/−*^ and *PARK2* ^*−/−*^ mice showed significantly increased dendritic cell frequencies compared to wild‐type mice in middle‐aged and aged groups (all *p* < 0.0001), with no significant differences between the two knockout genotypes. Overall, dendritic cell frequencies increased with age during the acute phase of EAE.

Additionally, a significant increase in CD11b^+^ CD115^+^ monocytes/macrophages was observed during EAE, with a clear age‐dependent pattern (Figure 3C1–C3). Two‐way ANOVA revealed significant effects of genotype and age, as well as a genotype × age interaction (*p* < 0.0001 for all factors). Post hoc Tukey's multiple comparisons test showed that no significant differences were detected between genotypes in young mice, whereas both *PINK1* ^*−/−*^ and *PARK2* ^*−/−*^ mice exhibited significantly higher frequencies of these myeloid cells compared to age‐matched wild‐type controls in middle‐aged and aged groups (all *p* < 0.0001), with no significant differences between the two knockout genotypes. These distinct immune profiles partly explain the differences in EAE progression across age groups in knockout mice. No significant differences were detected in peripheral immune cell populations between the sham‐immunized control groups (Figure 3A4,B4,C4).

To further characterize the functional activation state of T‐cell populations during EAE, intracellular cytokine staining was performed on splenocytes following ex vivo stimulation with PMA/ionomycin. Baseline IFN‐γ production in unstimulated cells was low across all genotypes. Following stimulation, flow cytometric analysis revealed a significant effect of genotype on the frequency of IFN‐γ‐producing CD4^+^ T cells (one‐way ANOVA, *F*(2,27) = 24.66, *p* < 0.0001). Both *PINK1* ^*−/−*^ and *PARK2* ^*−/−*^ mice exhibited significantly increased frequencies of CD4^+^IFN‐γ^+^ cells compared with age‐matched wild‐type controls (Figure 3D1–D4), indicating an enhanced Th1‐type inflammatory response associated with mitophagy deficiency.

Similarly, genotype significantly affected the frequency of IFN‐γ‐producing CD8+ T cells following stimulation (one‐way ANOVA, *F*(2,27) = 60.99, *p* < 0.0001). Both *PINK1* ^*−/−*^ and *PARK2* ^*−/−*^ mice showed significantly increased proportions of CD8^+^IFN‐γ^+^ cells compared with wild‐type animals (Figure 3E1–E4). Notably, the increase was more pronounced in the CD8^+^ compartment, suggesting enhanced activation of cytotoxic/inflammatory T‐cell responses during EAE. No significant differences were observed between the two knockout strains for either CD4^+^ or CD8^+^ IFN‐γ production. In contrast, sham‐immunized mice displayed low frequencies of IFN‐γ‐producing CD4^+^ and CD8^+^ T cells regardless of genotype.

These findings indicate that the increased abundance of peripheral T cells observed in mitophagy‐deficient mice is accompanied by enhanced pro‐inflammatory cytokine production, supporting the presence of an exacerbated pathogenic immune response during EAE.

### 3.3 Histological and Immunohistochemical Analysis of the Spinal Cord During the Acute Phase of EAE

Histological and immunohistochemical analyses revealed a marked expansion of inflammatory lesions in the spinal cord of MOG_35_–_55_‐immunized mice at peak disease (Figure 4A1–A4). Consistent with this, immunostaining with anti‐CD3 antibodies demonstrated a significantly higher number of infiltrating CD3^+^ T cells in the white matter of aged *PINK1* ^*−/−*^ and *PARK2* ^*−/−*^ mice compared to wild‐type controls (Figure 4B1–B4). Kruskal–Wallis analysis followed by Dunn's multiple comparisons test confirmed a significant increase in CD3^+^ T‐cell infiltration in both knockout genotypes compared to wild‐type mice (*p* = 0.0186 and *p* = 0.0328, respectively), with no significant differences between *PINK1* ^*−/−*^ and *PARK2* ^*−/−*^ mice.

**FIGURE 4:**
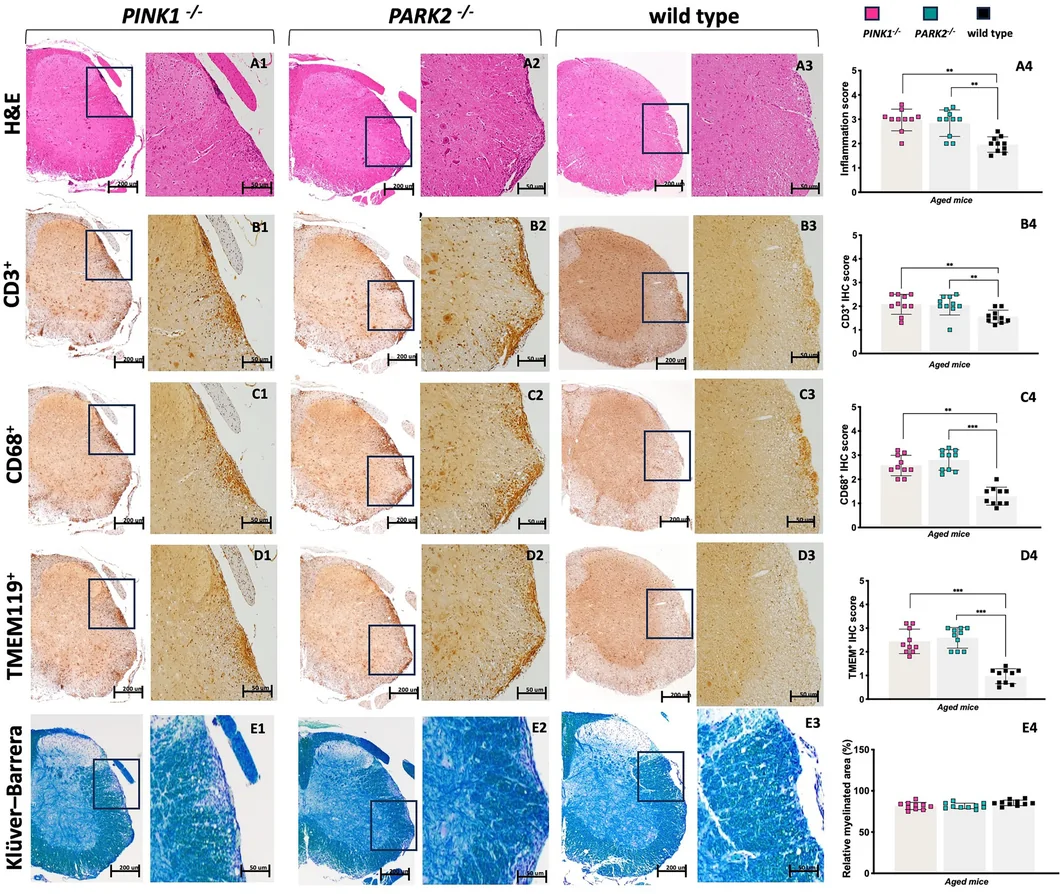
Histological and immunohistochemical analysis of the spinal cord during the acute phase of EAE. Representative lumbar spinal cord sections from aged wild type, *PINK1* ^*−/−*^, and *PARK2* ^*−/−*^ mice show inflammatory cell infiltration (hematoxylin and eosin staining) (A1–A3), presence of infiltrating CD3^+^ lymphocytes (B1–B3), CD68^+^ activated myeloid cells (C1–C3), TMEM119^+^ microglia in the white matter (D1–D3), and myelin staining assessed by the Klüver–Barrera method (E1–E3). Semi‐quantitative analysis (A4–D4) was performed by quantifying the area fraction of positive staining in white matter regions from four randomly selected sections per animal (*N* = 10 mice per group). Relative myelinated area was quantified from Klüver–Barrera‐stained sections (E4). Data are presented as mean ± standard error of the mean (SEM). Statistical analysis for panels A4–D4 was performed using the Kruskal–Wallis test followed by Dunn's multiple comparisons test. Panel E4 was analyzed using one‐way ANOVA followed by Tukey's multiple comparisons test. *p < 0.05; **p < 0.01; ***p < 0.001.

CD68 immunoreactivity revealed a substantial accumulation of activated myeloid cells in the white matter of aged *PINK1* ^*−/−*^ and *PARK2* ^*−/−*^ mice compared to wild‐type controls (Figure 4C1–C4). Kruskal–Wallis analysis followed by Dunn's multiple comparisons test confirmed a significant increase in CD68^+^ cells in both knockout genotypes (*p* = 0.0016 and *p* = 0.0001, respectively), with no significant differences between *PINK1* ^*−/−*^ and *PARK2* ^*−/−*^ mice.

Similarly, aged *PINK1* ^*−/−*^ and *PARK2* ^*−/−*^ mice exhibited a significantly higher density of TMEM119^+^ microglia in the white matter compared to age‐matched wild‐type controls (Figure 4D1–D4). Kruskal–Wallis analysis followed by Dunn's multiple comparisons test confirmed significantly increased TMEM119^+^ cell numbers in both knockout genotypes (*p* = 0.0007 and *p* = 0.0002, respectively), with no significant differences between *PINK1* ^*−/−*^ and *PARK2* ^*−/−*^ mice.

To evaluate whether the enhanced inflammatory response was associated with increased demyelination, spinal cord sections were stained using the Klüver–Barrera method (Figure 4E1–E4). Quantification of the relative myelinated area showed no significant differences between individual genotypes following Tukey's multiple comparisons test, despite a modest overall effect detected by one‐way ANOVA (*p* = 0.0484), indicating largely preserved myelin across groups. No significant differences in inflammation levels were observed between wild‐type, *PINK1* ^*−/−*^, and *PARK2* ^*−/−*^ mice under sham‐immunized conditions (data not shown).

### 3.4 Electron Microscopy

Transmission electron microscopy revealed genotype‐ and condition‐dependent alterations in mitochondrial morphology (Figure 5). In substantia nigra neurons, wild‐type mice consistently exhibited larger mitochondrial cross‐sectional area, whereas *PINK1* ^*−/−*^ and *PARK2* ^*−/−*^ mice displayed a higher mitochondrial number than wild‐type mice, although the magnitude of this effect varied across experimental conditions (Figure 5A–C). Two‐way ANOVA confirmed significant effects of condition (*F* = 218.6, *p* < 0.0001) and genotype (*F* = 118.9, *p* < 0.0001), with a significant interaction (*F* = 18.8, *p* < 0.0001). Post hoc analysis revealed increased mitochondrial number in both knockout genotypes compared to wild‐type mice in young and EAE conditions, while *PARK2* ^*−/−*^ mice showed the most pronounced increase in aged animals.

**FIGURE 5:**
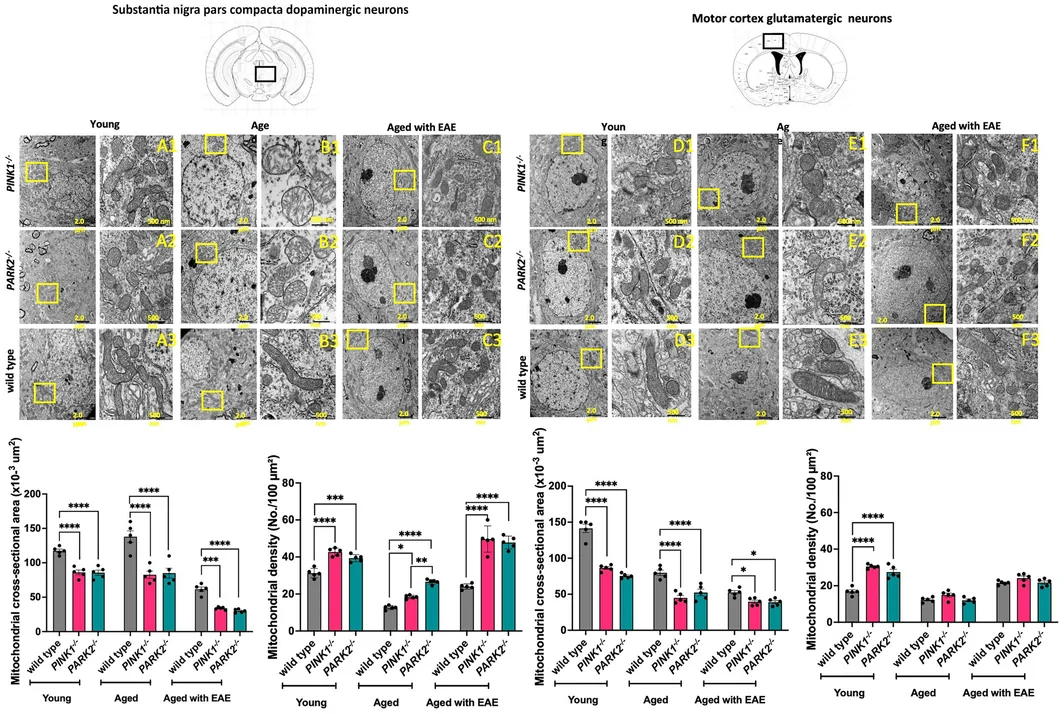
TEM analysis of mitochondrial ultrastructure in neurons of the substantia nigra pars compacta and motor cortex. Representative TEM images illustrate neuronal architecture and mitochondrial ultrastructure at low and high magnification in young, aged non‐EAE, and aged EAE WT, *PINK1* ^*−/−*^, and *PARK2* ^*−/−*^ mice. Yellow boxes in the low‐magnification images indicate the regions shown at higher magnification. Scale bars: 2 μm and 500 nm. Panels A–C show neurons from the substantia nigra pars compacta under young (A), aged non‐EAE (B), and aged EAE (C) conditions. Panels D–F show neurons from the motor cortex under young (D), aged non‐EAE (E), and aged EAE (F) conditions. Within each panel, rows 1, 2, and 3 correspond to *PINK1* ^*−/−*^, *PARK2* ^*−/−*^, and WT mice, respectively. Mitochondrial cross‐sectional area and numerical density were quantified in 20 cells per animal from five animals per group. Cell‐level measurements were averaged within each animal, and the resulting animal‐level means were used as independent biological replicates (*n* = 5 animals per group). Each dot represents one animal. Data are presented as mean ± SEM. Statistical analysis was performed using two‐way ANOVA, with genotype and experimental condition as factors, followed by Tukey's multiple‐comparisons test. *p < 0.05; **p < 0.01; ***p < 0.001; ****p < 0.0001.

Analysis of mitochondrial area in substantia nigra neurons showed significant effects of condition (*F* = 151.8, *p* < 0.0001) and genotype (*F* = 68.13, *p* < 0.0001), together with a significant genotype × condition interaction (*F* = 2.94, *p* = 0.0335). Post hoc analyses confirmed a persistent reduction in mitochondrial area in both *PINK1* ^*−/−*^ and *PARK2* ^*−/−*^ mice compared to wild‐type controls, although the magnitude of these differences varied across conditions (Figure 5D–F).

In cortical neurons, mitochondrial alterations were more restricted. Mitochondrial number was significantly influenced by condition (*F* = 131.8, *p* < 0.0001) and genotype (*F* = 33.16, *p* < 0.0001), with a significant interaction between the two factors (*F* = 14.74, *p* < 0.0001). Increased mitochondrial number in knockout mice was observed only in young animals, whereas no significant differences were detected in aged or EAE conditions (Figure 5G–I).

Similarly, mitochondrial area in cortical neurons showed significant effects of condition (*F* = 112.4, *p* < 0.0001) and genotype (*F* = 242.7, *p* < 0.0001), as well as a significant genotype × condition interaction (*F* = 19.35, *p* < 0.0001). While mitochondrial area was markedly reduced in both knockout strains compared with wild‐type mice under basal conditions, these differences became substantially smaller in aged EAE mice, indicating that genotype‐dependent differences in mitochondrial morphology were less pronounced under conditions of aging and neuroinflammation (Figure 5J–L).

### 3.5 PINK1 and PARK2 Deficiency Is Associated With Altered Mitochondrial Dynamics‐Related Proteins Profiles in Aged Mice

To further investigate the mitochondrial abnormalities observed in aged *PINK1* ^*−/−*^ and *PARK2* ^*−/−*^ mice, we examined key regulators of mitochondrial dynamics in midbrain mitochondrial fractions (Figure 6). Because non‐EAE and EAE samples were analyzed on separate membranes, densitometric and statistical analyses were performed independently within each condition, and no direct quantitative comparisons were made between non‐EAE and EAE samples.

**FIGURE 6:**
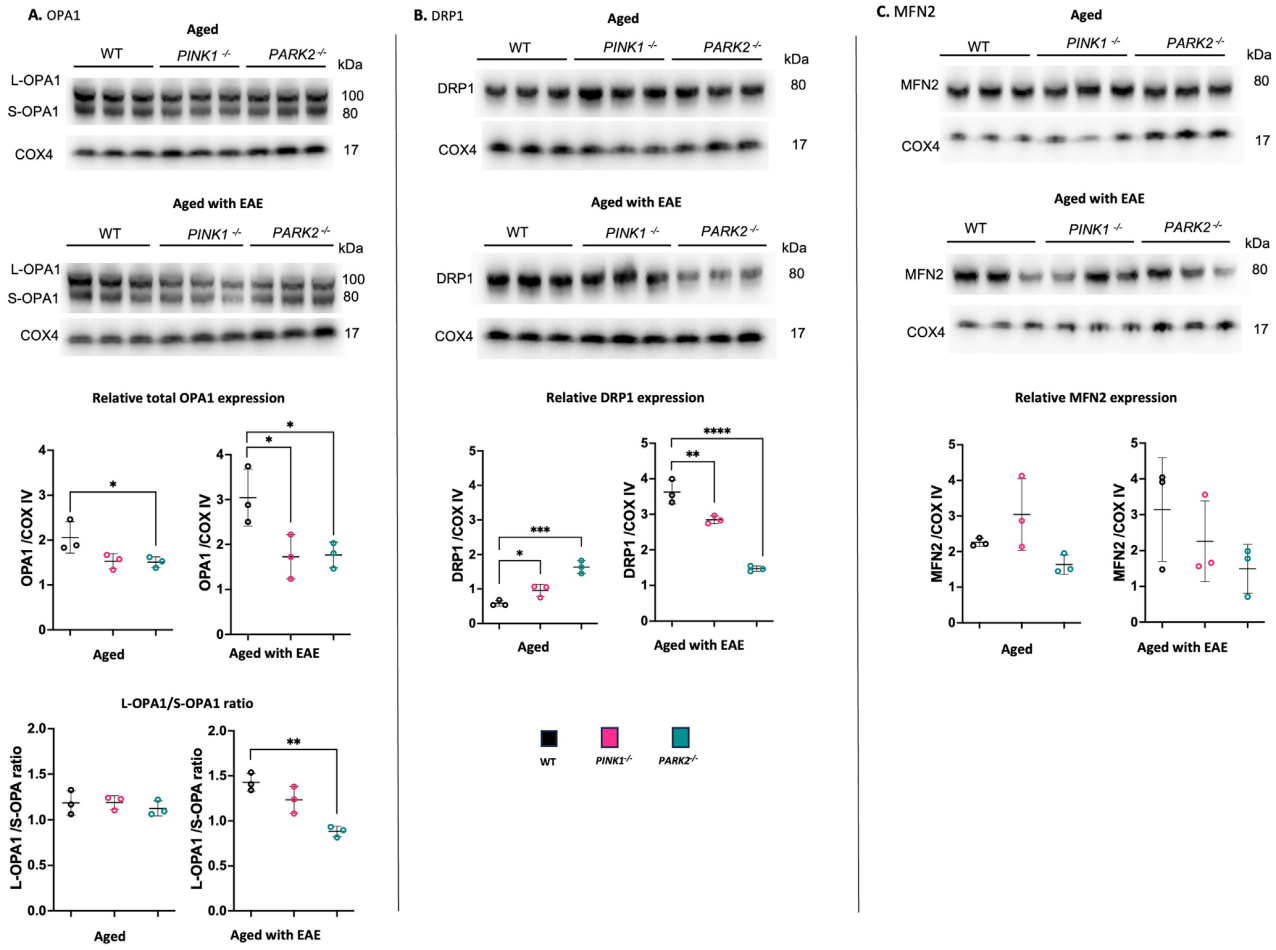
Alterations in mitochondrial dynamics‐associated proteins in aged *PINK1* ^*−/−*^ and *PARK2* ^*−/−*^ mice during EAE. (A) Representative Western blots and quantitative analysis of total OPA1 expression and the L‐OPA1/S‐OPA1 ratio in aged wild‐type (WT), *PINK1* ^*−/−*^, and *PARK2* ^*−/−*^ mice under non‐EAE conditions and at peak EAE. Total OPA1 was calculated as the combined L‐OPA1 and S‐OPA1 signal normalized to COX4. (B) Representative Western blots and quantitative analysis of DRP1 expression. (C) Representative Western blots and quantitative analysis of MFN2 expression. Protein levels were normalized to COX4. Non‐EAE and EAE samples were analyzed on separate membranes and were therefore quantified and statistically analyzed independently. Data are presented as individual values with mean ± SD (*n* = 3 mice/group). Statistical analysis was performed using ordinary one‐way ANOVA followed by Dunnett's multiple‐comparisons test, with WT as the reference group. *p < 0.05, **p < 0.01, ***p < 0.001, ****p < 0.0001.

In aged mice without EAE, total OPA1 expression differed significantly among genotypes (one‐way ANOVA, *F*(2,6) = 5.289, *p* = 0.0474) (Figure 6A). Dunnett's multiple‐comparisons test showed significantly lower OPA1 expression in *PARK2* ^*−/−*^ mice compared with WT mice (adjusted *p* = 0.0499), whereas the reduction in *PINK1* ^*−/−*^ mice did not reach statistical significance (adjusted *p* = 0.0566). The L‐OPA1/S‐OPA1 ratio was comparable among genotypes (*F*(2,6) = 0.3903, *p* = 0.6929). DRP1 expression, however, differed markedly among genotypes (*F*(2,6) = 35.02, *p* = 0.0005), with significantly higher levels in both *PINK1* ^*−/−*^ (adjusted *p* = 0.0464) and *PARK2* ^*−/−*^ mice (adjusted *p* = 0.0003) compared with WT mice (Figure 6B). MFN2 expression did not significantly differ among genotypes (*F*(2,6) = 4.043, *p* = 0.0773) (Figure 6C).

In aged mice with EAE, total OPA1 expression also differed significantly among genotypes (*F*(2,6) = 6.940, *p* = 0.0275), with significantly lower levels in both *PINK1* ^*−/−*^ (adjusted *p* = 0.0298) and *PARK2* ^*−/−*^ mice (adjusted *p* = 0.0337) compared with WT mice (Figure 6A). The L‐OPA1/S‐OPA1 ratio was also significantly different among genotypes (*F*(2,6) = 19.40, *p* = 0.0024), driven by a marked reduction in *PARK2* ^*−/−*^ mice compared with WT mice (adjusted *p* = 0.0015), whereas *PINK1* ^*−/−*^ mice did not significantly differ from WT (adjusted *p* = 0.1211). DRP1 expression showed a strong genotype effect (*F*(2,6) = 81.52, *p* < 0.0001), with significantly lower levels in *PINK1* ^*−/−*^ (adjusted *p* = 0.0069) and *PARK2* ^*−/−*^ mice (adjusted *p* < 0.0001) compared with WT mice (Figure 6B). MFN2 expression remained comparable among genotypes (*F*(2,6) = 1.603, *p* = 0.2768) (Figure 6C).

Together, these findings demonstrate genotype‐associated alterations in OPA1 and DRP1, while MFN2 remained relatively preserved, supporting a selective dysregulation of mitochondrial dynamics in aged *PINK1*‐ and *PARK2*‐deficient mice.

## 4 Discussion

This study demonstrates that PINK1 or Parkin deficiency shapes the clinical, immune, and mitochondrial response to autoimmune neuroinflammation in an age‐dependent manner. *PINK1* ^*−/−*^ and *PARK2* ^*−/−*^ mice developed a greater cumulative disease burden and incomplete clinical recovery, with the most pronounced phenotype observed in aged *PARK2* ^*−/−*^ mice. These clinical changes were accompanied by enhanced inflammatory‐cell infiltration, altered peripheral immune profiles, and marked changes in neuronal mitochondrial morphology. Collectively, our findings identify PINK1 and Parkin as important modulators of the interaction between immune aging and mitochondrial homeostasis during inflammatory stress.

Aging influenced different phases of EAE in distinct ways. Aged WT mice showed delayed disease onset, consistent with age‐associated immune changes in immune responsiveness and reduced induction of autoreactive T‐cell responses (Atkinson et al. 2022) (Wang et al. 2022). but did not exhibit clearly improved recovery. Immune aging may therefore attenuate or delay disease induction without promoting resolution once neuroinflammation has been established. Aging may simultaneously impair processes required for recovery, including immune‐cell contraction, debris clearance, remyelination, and restoration of tissue homeostasis. Its effects on EAE consequently appear to depend on the stage of disease rather than being uniformly protective or detrimental.

The immune phenotype represents one of the most robust findings of the present study. Knockout mice exhibited altered dendritic‐cell and monocyte populations and increased frequencies of CD8^+^ T cells. Intracellular cytokine analyses additionally demonstrated increased frequencies of IFN‐γ‐producing CD4^+^ and CD8^+^ T cells in aged *PINK1* ^*−/−*^ and *PARK2* ^*−/−*^ mice with EAE following ex vivo stimulation. These results support an enhanced pro‐inflammatory T‐cell phenotype associated with loss of PINK1 or Parkin and extend previous evidence linking these proteins to peripheral and central immune regulation (Cossu et al. 2022; Cossu, Yokoyama, Sato, et al. 2021). Alterations in myeloid and T‐cell populations were also evident in middle‐aged knockout mice, indicating that immune dysregulation develops before advanced aging. This finding is relevant to neuroinflammatory conditions in which CD8^+^ T‐cell responses have been associated with tissue injury and disease progression (Ma et al. 2025; Salou et al. 2015; von Essen et al. 2023).

MOG‐specific T‐cell proliferation followed a non‐linear age‐dependent pattern, with robust responses in young mice, attenuation in middle‐aged animals, and re‐emergence of genotype‐associated differences in aged mice. The middle‐aged attenuation may reflect a transitional phase of immune aging involving changes in T‐cell repertoire, proliferative capacity, activation thresholds, and metabolic fitness (Nikolich‐Zugich 2018). The re‐emergence of genotype‐associated differences in aged mice does not necessarily indicate restoration of immune competence. Instead, it may reflect the interaction between progressive immunosenescence, age‐associated low‐grade inflammation, and the cumulative consequences of PINK1 or Parkin deficiency. Under these conditions, reduced mitochondrial and metabolic adaptability may become increasingly important in determining how immune cells respond to antigenic stimulation.

PINK1 and Parkin are expressed in multiple immune‐cell populations and participate in mitochondrial homeostasis, inflammatory signaling, antigen presentation, and T‐cell function (Matheoud et al. 2016). The changes in dendritic cells, monocytes, CD8^+^ T cells, and IFN‐γ‐producing lymphocytes therefore support an important contribution of the immune compartment to the aggravated clinical phenotype. This broadens the biological significance of PINK1 and Parkin beyond their established neuronal functions and suggests that their loss may alter the capacity of both peripheral and CNS‐resident cells to respond appropriately to inflammatory stress.

EAE was used as a neuroinflammatory challenge capable of generating peripheral immune activation and CNS inflammatory stress. Neurons within the substantia nigra pars compacta were examined as a metabolically demanding and mitochondria‐dependent population potentially sensitive to altered mitochondrial homeostasis (Wang et al. 2023), rather than as a primary pathological target of EAE. Motor cortex neurons were included to assess whether the mitochondrial phenotype was confined to the substantia nigra or shared across neuronal populations. Ultrastructural analysis revealed increased mitochondrial number and reduced mitochondrial cross‐sectional area in knockout neurons, a morphological pattern consistent with increased fragmentation. These alterations were particularly evident in substantia nigra neurons and were also detected, although less uniformly, in the motor cortex. This regional pattern suggests that the mitochondrial response to PINK1 or Parkin deficiency varies according to neuronal context, metabolic demand, age, and inflammatory status. The more consistent alterations observed in substantia nigra neurons are compatible with the recognized sensitivity of this population to mitochondrial perturbation (Wang et al. 2023).

The biochemical analyses further support an alteration of mitochondrial homeostasis. Under non‐EAE conditions, total OPA1 was reduced in *PARK2* ^*−/−*^ mice, whereas DRP1 abundance was increased in both knockout strains. Within the EAE group, total OPA1 was reduced in both *PINK1* ^*−/−*^ and *PARK2* ^*−/−*^ mice, the L‐OPA1/S‐OPA1 ratio was reduced in *PARK2* ^*−/−*^ mice, and DRP1 abundance was lower in both knockout strains relative to WT mice. MFN2 remained unchanged under both experimental conditions. Because non‐EAE and EAE samples were analyzed on separate membranes, these patterns cannot be interpreted as direct EAE‐induced changes. Nevertheless, the selective and opposing patterns of OPA1 and DRP1 do not indicate a simple constitutive shift toward either fission or fusion. Instead, they suggest that mitochondrial responses to PINK1 or Parkin deficiency depend on the physiological and inflammatory context.

Rather than causing a major and uniform alteration in mitochondrial dynamics under baseline conditions, PINK1 or Parkin deficiency may become particularly consequential when mitochondria are challenged by inflammatory, metabolic, or age‐associated stress. In this model, loss of PINK1 or Parkin reduces mitochondrial adaptability and limits the capacity of cells to maintain homeostasis as demand increases. This stress‐dependent vulnerability provides a coherent interpretation of why mitochondrial abnormalities and clinical differences become more prominent during aging and EAE. It may also be conceptually relevant to *PARK2*‐associated disease, which is characterized by early onset but often a relatively prolonged clinical course (Menon et al. 2024) (Dawson and Dawson 2010). Parkin deficiency may not produce catastrophic mitochondrial dysfunction by itself but may reduce the capacity of susceptible cells to accommodate additional stress over time. At peak disease, increased spinal cord inflammatory infiltration in knockout mice was not accompanied by significant genotype‐dependent differences in Klüver–Barrera myelin staining. This suggests that the enhanced inflammatory phenotype did not translate into detectable differences in myelin loss at this disease stage.

Although EAE is not a model of PD, the findings illustrate how alterations in mitochondrial homeostasis may influence the response to inflammation across disease contexts. In MS, CD8^+^ T cells contribute to gray‐matter pathology and progressive disability (Salou et al. 2015), whereas in PD, mitochondrial stress and chronic inflammation converge on vulnerable neuronal populations (Ma et al. 2025). Mutations in *PINK1* and *PARK2* cause early‐onset PD (Valente et al. 2004) (Dawson and Dawson 2010) (Quinn et al. 2020), but the functions of these pathways extend beyond monogenic neurodegeneration. Chronic microglial activation, altered cytokine profiles, and peripheral immune involvement are increasingly recognized in PD pathogenesis (Cossu et al. 2023), while detection of PINK1 and Parkin proteins in serum and CSF during MS relapse suggests potential relevance to neuroinflammatory activity (Cossu, Yokoyama, Sechi, and Hattori 2021). Together, these observations support a broader model in which PINK1 and Parkin contribute to cellular adaptation to inflammatory and metabolic stress.

This study has several limitations. All experiments were conducted in female mice, and potential sex‐dependent effects remain to be examined. Tissue analyses were restricted to peak disease and were performed in cohorts independent from those monitored clinically until Day 39; therefore, the pathological basis of incomplete clinical recovery could not be determined. Axonal injury, oligodendrocyte survival, remyelination, T‐cell exhaustion and cytotoxicity, and APC function were not directly assessed. Electron microscopy and analysis of OPA1, DRP1, and MFN2 do not directly measure mitochondrial function, mitophagic flux, or functional fusion and fission rates. Finally, the systemic knockout models do not allow the relative contributions of immune cells, neurons, and other CNS‐resident populations to be separated. Future studies incorporating both sexes, cell‐specific models, functional mitochondrial assays, and recovery or chronic disease time points will be required to address these questions. In conclusion, PINK1 or Parkin deficiency aggravates the clinical and inflammatory course of EAE in aging and is accompanied by marked alterations in neuronal mitochondrial morphology and dynamics‐related proteins. Rather than causing a uniform disruption of mitochondrial dynamics under baseline conditions, PINK1 or Parkin deficiency may become particularly consequential during inflammatory or metabolic stress, reducing the capacity of cells to adapt appropriately. These findings identify PINK1 and Parkin as important regulators at the interface between aging, immunity, mitochondrial homeostasis, and neuroinflammation.

## Author Contributions

**Davide Cossu:** data curation, methodology, writing – review and editing, writing – original draft, funding acquisition, investigation. **Sachiko Noda:** methodology, data curation. **Shigeto Sato:** methodology. **Kazumasa Yokoyama:** formal analysis, data curation. **Yuji Tomizawa:** formal analysis, data curation. **Ruochen Wang:** data curation, formal analysis. **Sarah Vascellari:** data curation, formal analysis. **Leonardo Antonio Sechi:** data curation, formal analysis. **Taku Hatano:** supervision, writing – review and editing. **Nobutaka Hattori:** supervision, conceptualization, writing – review and editing.

## Funding

This research was funded by the Italian Ministry of University and Research (MUR) under the National Recovery and Resilience Plan (PNRR), funded by the European Union – NextGenerationEU, Mission 4, Component 2, Investment 1.1 (PRIN), CUP I53D23000970006; and by JSPS KAKENHI Grant Number 24H00068 to HN. All other authors report no financial disclosures relevant to this manuscript.

## Ethics Statement

All animal procedures were conducted in accordance with institutional and national guidelines and were approved by the Institutional Animal Care and Use Committee of Juntendo University School of Medicine (approval No. 290238), as detailed in the Materials and Methods section.

## Conflicts of Interest

The authors declare no competing financial interests.

## Supporting information


**Table S1:** List of the primary and secondary antibodies.
**Table S2:** Assessment of data normality. Data distribution was evaluated using the Shapiro–Wilk test. *p*‐values are reported for each dataset. A *p*‐value ≤ 0.05 was considered indicative of non‐normal distribution.
**Table S3:** Full statistical reporting of all analyses.
**Figure S1:** Full‐length western blot images corresponding to Figure 6.

## Data Availability

The datasets generated and/or analyzed during the current study are available from the corresponding author upon reasonable request.
