# Supplementary material for: PINK1 and PARKIN Deficiency Increases Neuronal Vulnerability to Autoimmune Neuroinflammation Through Mitochondrial Fragmentation in Aged Mice
Source: J Neurochem. 2026 Oct 23;170(10):e70561. doi: 10.1111/jnc.70561 (PMC13691613; doi:10.1111/jnc.70561)

# PINK1 and PARKIN deficiency increases neuronal vulnerability to autoimmune neuroinflammation through mitochondrial fragmentation in aged mice

**Davide Cossu[1,2,3, †], Sachiko Noda[1,4, †], Shigeto Sato[1,5], Kazumasa Yokoyama[1], Yuji Tomizawa[1], Ruochen Wang[1], Sarah Vascellari[6], Leonardo Antonio Sechi[3,7], Taku Hatano[1], Nobutaka Hattori[1,4,8, *]**

[1] Juntendo University, Department of Neurology, Tokyo, Japan

[2] Juntendo University, Biomedical Research Core Facilities, Tokyo, Japan

[3] Sassari University, Department of Biomedical Sciences, Sassari, Italy

[4] Juntendo University, Neuron-Glia Crosstalk center, Tokyo, Japan

[5] Juntendo University, Center for Biomedical Research Resources, Tokyo, Japan

[6] Cagliari University, Department of Biomedical Sciences, Cagliari, Italy

[7] SC Microbiologia Azienda Ospedaliero Universitaria, Sassari, Italy

[8] RIKEN Center for Brain Science, Neurodegenerative Disorders Collaborative Laboratory, Wako, Japan

*** Correspondence:**
Nobutaka Hattori
nhattori@juntendo.ac.jp

**Supplementary Table 1.** List of the primary and secondary antibodies

| Antigen | Clone | Technique | Dilution | Catalog no | RRID | Supplier |
|---|---|---|---|---|---|---|
| **Primary antibodies** | | | | | | |
| anti-mouse CD16/CD32 | 93 | Flow Cyt | 1:100 | 101302 | RRID:AB_312801 | BioLegend |
| FITC anti-mouse CD4 | GK1.5 | Flow Cyt | 1:200 | 100406 | RRID:AB_312691 | BioLegend |
| Brilliant Violet 421™ anti-mouse CD8a | 53-6.7 | Flow Cyt | 1:100 | 100738 | RRID:AB_11218778 | BioLegend |
| PerCP/Cyanine5.5 anti-mouse CD3 | 17A2 | Flow Cyt | 1:100 | 100218 | RRID:AB_312673 | BioLegend |
| Alexa Fluor® 488 anti-mouse CD11c | N418 | Flow Cyt | 1:50 | 117313 | RRID:AB_312673 | BioLegend |
| PE/Cyanine7 anti-mouse CD19 | 1D3 | Flow Cyt | 1:100 | 152418 | RRID:AB_2616891 | BioLegend |
| Alexa Fluor® 700 anti-mouse Ly-6G | 1A8 | Flow Cyt | 1:100 | 127622 | RRID:AB_10640816 | BioLegend |
| PE/Cyanine7 anti-mouse/human CD11b | M1/70 | Flow Cyt | 1:100 | 101216 | RRID:AB_10640816 | BioLegend |
| APC anti-mouse CD115 | AFS98 | Flow Cyt | 1:100 | 135506 | RRID:AB_312799 | BioLegend |
| Brilliant Violet 510™ anti-mouse I-A/I-E | M5/114.15.2 | Flow Cyt | 1:100 | 107635 | RRID:AB_2565860 | BioLegend |
| PE anti-mouse IFN-γ | XMG1.2 | Flow Cyt | 1:100 | 505808 | RRID: AB_315402 | BioLegend |
| Anti- CD3 (rabbit polyclonal) | | IHC-P | 1:100 | ab16044 | RRID:AB_2565860 | Abcam |
| Anti-CD68 (rabbit polyclonal) | | IHC-P | 1:500 | ab283654 | RRID:AB_2889189 | Abcam |
| Anti-TMEM119(rabbit monoclonal) | | IHC-P | 1:300 | ab209064 | RRID:AB_2889189 | Abcam |
| OPA1 (rabbit monoclonal) | D6U6N | WB | 1:1000 | 80471 | RRID:AB_2734117 | Cell Signaling Technology |
| MFN2 (rabbit monoclonal) | D2D10 | WB | 1:1000 | 9482 | RRID:AB_2716838 | Cell Signaling Technology |
| DRP1 (rabbit monoclonal) | D6C7 | WB | 1:1000 | 8570 | RRID:AB_10950498 | Cell Signaling Technology |
| COXIV (rabbit polyclonal) | | WB | 1:5000 | 11242-1-AP | RRID:AB_2085278 | Proteitech |
| **Secondary antibodies** | | | | | | |
| Goat anti-Rabbit IgG (H+L) Secondary Antibody, Biotin | | IHC-P | 1:1,000 | 65-6140 | RRID:AB_2533939 | Invitrogen |
| Goat anti-Rabbit IgG (H+L) Alexa Fluor™ 488 | | IF | 1:1,000 | A-11008 | RRID:AB_143165 | Invitrogen |

**Supplementary Table 2. Assessment of data normality.** Data distribution was evaluated using the Shapiro–Wilk test. P-values are reported for each dataset. A p-value ≤ 0.05 was considered indicative of non-normal distribution.

| **Dataset** | **Variable** | **Test** | **p-value** | **Distribution** |
|---|---|---|---|---|
| Clinical score | Daily scores | Shapiro–Wilk | <0.0001 | Non-normal |
| Clinical score | Peak score | Shapiro–Wilk | 0.02 | Non-normal |
| Clinical score | Cumulative score | Shapiro–Wilk | 0.07 | Normal |
| Body weight | % change | Shapiro–Wilk | 0.001 | Non-normal |
| Flow cytometry | $CD8^+$ T cells | Shapiro–Wilk | 0.80 | Normal |
| Flow cytometry | Dendritic cells | Shapiro–Wilk | 0.11 | Normal |
| Flow cytometry | Monocytes | Shapiro–Wilk | 0.90 | Normal |
| Flow cytometry | INF-γ T cells | Shapiro–Wilk | 0.60 | Normal |
| T cell proliferation | CPM / index | Shapiro–Wilk | 0.80 | Normal |
| Histology | H&E score | Shapiro–Wilk | 0.10 | Normal |
| Histology | Klüver–Barrera | Shapiro–Wilk | 0.44 | Normal |
| IHC | $CD3^+$ cells | Shapiro–Wilk | 0.01 | Non-normal |
| IHC | $CD68^+$ cells | Shapiro–Wilk | 0.04 | Non-normal |
| IHC | $TMEM119^+$ cells | Shapiro–Wilk | 0.02 | Non-normal |
| TEM | Mitochondrial number | Shapiro–Wilk | 0.20 | Normal |
| TEM | Mitochondrial area | Shapiro–Wilk | 0.13 | Normal |

## Supplementary Table 3. Full statistical reporting of all analyses.

| Figure/Panel | Dataset | Age group | Test | Overall Statistic | df | Overall p-value | Post hoc |
|---|---|---|---|---|---|---|---|
| Fig.2 A1 | Clinical score kinetics | Young | Two-way ANOVA | F = 32.30 (time); F = 6.523 (genotype); F = 0.8552 (interaction) | (38,1623); (2,1623); (76,1623) | p < 0.0001 | Tukey: WT vs PINK1$^{-/-}$ p < 0.0001; WT vs PARK2$^{-/-}$ p < 0.0001; PINK1$^{-/-}$ vs PARK2$^{-/-}$ p < 0.0001 |
| Fig.2 A2 | Clinical score kinetics | Middle-aged | Two-way ANOVA | F = 34.83 (time); F = 149.6 (genotype); F = 6.695 (interaction) | (38,1638); (2,1638); (76,1638) | p < 0.0001 | Tukey: WT vs PINK1$^{-/-}$ p < 0.0001; WT vs PARK2$^{-/-}$ p < 0.0001; PINK1$^{-/-}$ vs PARK2$^{-/-}$ p < 0.0001 |
| Fig.2 A3 | Clinical score kinetics | Aged | Two-way ANOVA | F = 28.72 (time); F = 57.97 (genotype); F = 3.250 (interaction) | (41,1764); (2,1764); (82,1764) | p < 0.0001 | Tukey: WT vs PINK1$^{-/-}$ p < 0.0001; WT vs PARK2$^{-/-}$ p < 0.0001; PINK1$^{-/-}$ vs PARK2$^{-/-}$ p < 0.0001 |
| Fig.2 B1 | Cumulative score | Young | Kruskal–Wallis | H = 4.731 | df = 2 | p = 0.09 | ns |
| Fig.2 B2 | Cumulative score | Middle-aged | Kruskal–Wallis | H = 5.993 | df = 2 | p =0.05 | Dunn: WT vs PINK1$^{-/-}$ p = 0.0433 |
| Fig.2 B3 | Cumulative score | Aged | Kruskal–Wallis | H = 3.809 | df = 2 | p = 0.15 | ns |
| Fig.2 C1 | Peak score | Young | Kruskal–Wallis | H = 20.94 | df = 2 | p < 0.0001 | Dunn: WT vs PARK2$^{-/-}$ p < 0.0001; PINK1$^{-/-}$ vs PARK2$^{-/-}$ p = 0.0007 |
| Fig.2 C2 | Peak score | Middle-aged | Kruskal–Wallis | H = 32.38 | df = 2 | p < 0.0001 | Dunn: WT vs PARK2$^{-/-}$ p < 0.0001; PINK1$^{-/-}$ vs PARK2$^{-/-}$ p = 0.0009 |
| Fig.2 C3 | Peak score | Aged | Kruskal–Wallis | H = 23.49 | df = 2 | p < 0.0001 | Dunn: WT vs PARK2$^{-/-}$ p < 0.0001; PINK1$^{-/-}$ vs PARK2$^{-/-}$ p = 0.0297 |
| Fig.2 D1 | Body weight | Young | Two-way ANOVA | F = 52.20 (time); F = XX (genotype); F = 6.400 (interaction) | (38,1638); (2,1638); (76,1638) | p < 0.0001 | Tukey: WT vs PINK1$^{-/-}$ p < 0.0001; WT vs PARK2$^{-/-}$ p < 0.0001; PINK1$^{-/-}$ vs PARK2$^{-/-}$ p < 0.0001 |
| Fig.2 D2 | Body weight | Middle-aged | Two-way ANOVA | F = 104.5 (time); F = 400.9 (genotype); F = 8.80 (interaction) | (38,1638); (2,1638); (76,1638) | p < 0.0001 | Tukey: WT vs PINK1$^{-/-}$ p<0.0001; WT vs PARK2$^{-/-}$ p<0.0001; PINK1$^{-/-}$ vs PARK2$^{-/-}$ ns |
| Fig.2 D3 | Body weight | Aged | Two-way ANOVA | F = 133.7 (time); F = 590.1 (genotype); F = 9.902 (interaction) | (38,1638); (2,1638); (76,1638) | p < 0.0001 | Tukey: WT vs PINK1$^{-/-}$ p < 0.0001; WT vs PARK2$^{-/-}$ p < 0.0001; PINK1$^{-/-}$ vs PARK2$^{-/-}$ p < 0.0001 |
| Fig.2 E1 | T cell proliferation | Young | Two-way ANOVA | Interaction F(2.317, 48.66) = 7.255 | | p = 0.0011 | Tukey; WT vs Parkin: MOG35–55 p < 0.0001, OVA p < 0.0001; PINK1 vs Parkin: MOG35–55 p < 0.0001, OVA p = 0.0252 |
| Fig.2 E2 | T cell proliferation | Middle-aged | Two-way ANOVA | Interaction: F(2.162, 45.40) = 5.049 | | p = 0.0607 | ns |
| Fig.2 E3 | T cell proliferation | Aged | Two-way ANOVA | Interaction: F(2.556, 53.6) = 7.965 | | p < 0.0001 | Tukey; multiple differences across conditions |
| Fig.3 A3 | Flow citometry CD8$^{+}$ cells | Young, millde-aged, aged | Two-way ANOVA | F = 17.84 (age); F = 128.8 (genotype); F = 6.22 (interaction) | (2,126); (2,126); (4,126) | p < 0.0001 | Tukey: WT vs PINK1$^{-/-}$ (young) p<0.0001; WT vs PARK2$^{-/-}$ (young) p<0.0001; PINK1$^{-/-}$ vs PARK2$^{-/-}$ (young) ns; WT vs PINK1$^{-/-}$ (middle-aged) p<0.0001; WT vs PARK2$^{-/-}$ (middle-aged) p<0.0001; PINK1$^{-/-}$ vs PARK2$^{-/-}$ (middle-aged) ns; |

| Figure/Panel | Dataset | Age group | Test | Overall Statistic | df | Overall p-value | Post hoc |
|---|---|---|---|---|---|---|---|
| | | | | | | | WT vs PINK1⁻/⁻ (aged) p<0.0001; WT vs PARK2⁻/⁻ (aged) p<0.0001; PINK1⁻/⁻ vs PARK2⁻/⁻ (aged) ns |
| Fig.3 B3 | Flow citometry Dendritic cells | Young,millde-aged, aged | Two-way ANOVA | F = 15.12 (age); F = 92.91 (genotype); F = 17.87 (interaction) | (2,126); (2,126); (4,126) | p < 0.0001 | Tukey: WT vs PINK1⁻/⁻ (young) p=0.0051; WT vs PARK2⁻/⁻ (young) ns; PINK1⁻/⁻ vs PARK2⁻/⁻ (young) ns; WT vs PINK1⁻/⁻ (middle-aged) p<0.0001; WT vs PARK2⁻/⁻ (middle-aged) p<0.0001; PINK1⁻/⁻ vs PARK2⁻/⁻ (middle-aged) ns; WT vs PINK1⁻/⁻ (aged) p<0.0001; WT vs PARK2⁻/⁻ (aged) p<0.0001; PINK1⁻/⁻ vs PARK2⁻/⁻ (aged) ns |
| Fig.3 C3 | Flow citometry monocytes | Young,millde-aged, aged | Two-way ANOVA | F = 244.3 (age); F = 179.0 (genotype); F = 48.93 (interaction) | (2,126); (2,126); (4,126) | p < 0.0001 | Tukey: WT vs PINK1⁻/⁻ (young) ns; WT vs PARK2⁻/⁻ (young) ns; PINK1⁻/⁻ vs PARK2⁻/⁻ (young) ns; WT vs PINK1⁻/⁻ (middle-aged) p<0.0001; WT vs PARK2⁻/⁻ (middle-aged) p<0.0001; PINK1⁻/⁻ vs PARK2⁻/⁻ (middle-aged) ns; WT vs PINK1⁻/⁻ (aged) p<0.0001; WT vs PARK2⁻/⁻ (aged) p<0.0001; PINK1⁻/⁻ vs PARK2⁻/⁻ (aged) ns |
| Fig.3 D3 | Flow citometry INF-γ CD4⁺ | Aged | One-way ANOVA | F = 24.66 | (2,24) | p < 0.0001 | Tukey: WT vs PINK1⁻/⁻ p=0.0002; WT vs PARK2⁻/⁻ p<0.0001; PINK1⁻/⁻ vs PARK2⁻/⁻ ns |
| Fig.3 E3 | Flow citometry INF-γ CD8⁺ | Aged | One-way ANOVA | F = 60.99 | (2,24) | p < 0.0001 | Tukey: WT vs PINK1⁻/⁻ p<0.0001; WT vs PARK2⁻/⁻ p<0.0001; PINK1⁻/⁻ vs PARK2⁻/⁻ ns |
| Fig.4 A4 | H&E / inflammatory infiltration | Aged | Kruskal–Wallis | H = 14.44 | df = 2 | p = 0.0007 | Dunn: WT vs PINK1⁻/⁻ p=0.0014; WT vs PARK2⁻/⁻ p=0.0074; PINK1⁻/⁻ vs PARK2⁻/⁻ ns |
| Fig.4 B4 | CD3⁺ lymphocytes | Aged | Kruskal–Wallis | H = 9.34 | df = 2 | p = 0.0094 | Dunn: WT vs PINK1⁻/⁻ p=0.0186; WT vs PARK2⁻/⁻ p=0.0328; PINK1⁻/⁻ vs PARK2⁻/⁻ ns |
| Fig.4 C4 | CD68⁺ myeloid cells | Aged | Kruskal–Wallis | H = 19.61 | df = 2 | p < 0.0001 | Dunn: WT vs PINK1⁻/⁻ p=0.0016; WT vs PARK2⁻/⁻ |

| Figure/Panel | Dataset | Age group | Test | Overall Statistic | df | Overall p-value | Post hoc |
|---|---|---|---|---|---|---|---|
| | | | | | | | p=0.0001; PINK1⁻/⁻ vs PARK2⁻/⁻ ns |
| Fig.4 D4 | TMEM119⁺ microglia | Aged | Kruskal–Wallis | H = 19.62 | df = 2 | p < 0.0001 | Dunn: WT vs PINK1⁻/⁻ p=0.0007; WT vs PARK2⁻/⁻ p=0.0002; PINK1⁻/⁻ vs PARK2⁻/⁻ ns |
| Fig.4 E4 | Klüver–Barrera Demyelination | Aged | One-way ANOVA | F = 3.396 | (2,27) | p = 0.048 | Tukey: WT vs PINK1⁻/⁻ ns; WT vs PARK2⁻/⁻ ns; PINK1⁻/⁻ vs PARK2⁻/⁻ ns |
| Fig.5 (SN) | Mitochondrial number | Young, aged, aged EAE | Two-way ANOVA | F = 218.6 (condition); F = 118.9 (genotype); F = 18.8 (interaction) | (2,36); (2,36); (4,36); | p < 0.0001 | Tukey: **Young:** WT vs PINK1 p<0.0001; WT vs PARK2 p=0.0005; PINK1 vs PARK2 ns **Aged:** WT vs PINK1 p=0.0136; WT vs PARK2 p<0.0001; PINK1 vs PARK2 p=0.0004; WT vs PINK1 ns **Aged EAE:** WT vs PINK1 p<0.0001; WT vs PARK2 p<0.0001; PINK1 vs PARK2 ns |
| Fig.5 (SN) | Mitochondrial area | Young, aged, aged EAE | Two-way ANOVA | F = 151.8 (condition); F = 68.13 (genotype); F = 2.94 (interaction, ns) | (2,36); (2,36); (4,36); | p < 0.0001 (condition, genotype); p = 0.0335 (interaction) | Tukey: **Young:** WT vs PINK1 p<0.0001; WT vs PARK2 p<0.0001; PINK1 vs PARK2 ns A**ged:** WT vs PINK1 p<0.0001; WT vs PARK2 p<0.0001; PINK1 vs PARK2 ns **Aged EAE:** WT vs PINK1 p=0.0005; WT vs PARK2 p<0.0001; PINK1 vs PARK2 ns |
| Fig.5 (cortex) | Mitochondrial number | Young, aged, aged EAE | Two-way ANOVA | F = 131.8 (condition); F = 33.16 (genotype); F = 14.74 (interaction) | (2,36); (2,36); (4,36); | p < 0.0001 | Tukey: **Young:** WT vs PINK1 p<0.0001; WT vs PARK2 p<0.0001; PINK1 vs PARK2 ns **Aged:** ns **Aged EAE:**ns |
| Fig.5 (cortex) | Mitochondrial | Young, aged, | Two-way | F = 242.7 (condition); | (2,36); | p < 0.0001 | Tukey: **Young:** WT vs |

| Figure/Panel | Dataset | Age group | Test | Overall Statistic | df | Overall p-value | Post hoc |
|---|---|---|---|---|---|---|---|
| | area | aged EAE | ANOVA | F = 112.4 (genotype); F = 19.35 (interaction) | (2,36); (4,36); | | PINK1 p<0.0001; WT vs PARK2 p<0.0001; PINK1 vs PARK2 ns **Aged:** WT vs PINK1 p<0.0001; WT vs PARK2 p<0.0001; PINK1 vs PARK2 ns **Aged EAE:** WT vs PINK1 p=0.0225; WT vs PARK2 p=0.0164; PINK1 vs PARK2 ns |
| Fig.6A | OPA1 | Aged | One-way ANOVA | F = 5.289 | (2,6) | p=0.0474 | Dunnett: WT vs PINK1 *p* = 0.0566; WT vs PARK2 *p* = 0.0499 |
| Fig.6A | OPA1 | Aged EAE | One-way ANOVA | F = 6.940 | (2,6) | p=0.0474 | Dunnett: WT vs PINK1 *p* = 0.0298; WT vs PARK2 *p* = 0.0337 |
| Fig.6A | L-OPA1/S-OPA1 ratio | EAE | One-way ANOVA | F = 0.3903 | (2,6) | p=0.06929 | Dunnett: WT vs PINK1 *p* = 0.9981; WT vs PARK2 *p* = 0.6969 |
| Fig.6A | L-OPA1/S-OPA1 ratio | Aged EAE | One-way ANOVA | F = 19.40 | (2,6) | *p*=0.0024 | Dunnett: WT vs PINK1 *p* = 0.1211; WT vs PARK2 *p* = 0.0015 |
| Fig.6B | DRP1 | Aged | One-way ANOVA | F = 35.02 | (2,6) | *p*=0.0005 | Dunnett: WT vs PINK1 *p* = 0.0464; WT vs PARK2 *p* = 0.0003 |
| Fig.6B | DRP1 | Aged EAE | One-way ANOVA | F = 81.52 | (2,6) | *p*<0.0001 | Dunnett: WT vs PINK1 *p* = 0.0069; WT vs PARK2 *p* < 0.0001 |
| Fig.6C | MFN2 | EAE | One-way ANOVA | F = 4.043 | (2,6) | *p*=0.0773 | Dunnett: WT vs PINK1 *p* = 0.2690; WT vs PARK2 *p* = 0.4026 |
| Fig.6C | MFN2 | Aged EAE | One-way ANOVA | F = 1.603 | (2,6) | *p*=0.2768 | Dunnett: WT vs PINK1 *p* = 0.5637; WT vs PARK2 *p* = 0.2054 |

**Supplementary Figure S1. Full-length Western blot images corresponding to Figure 6.**

**A. OPA1**

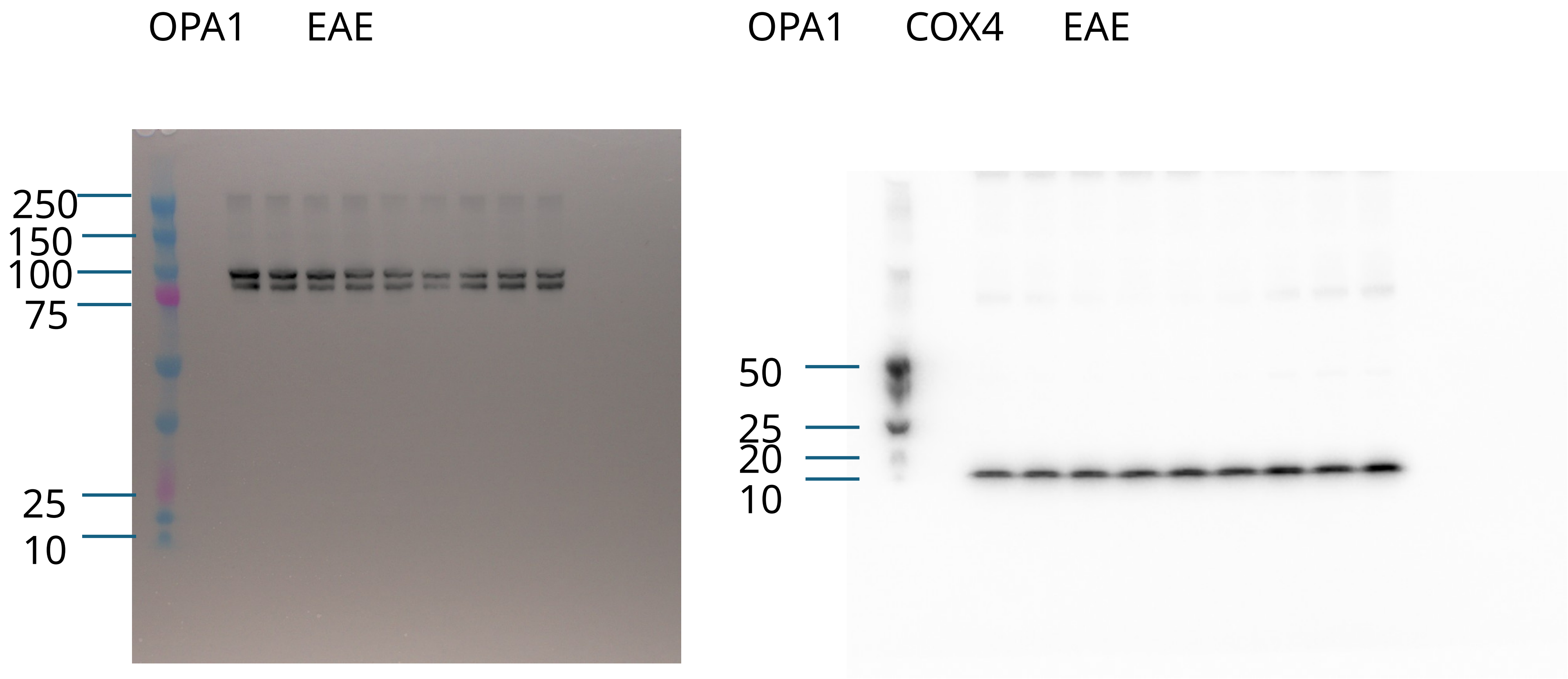

**A. OPA1**

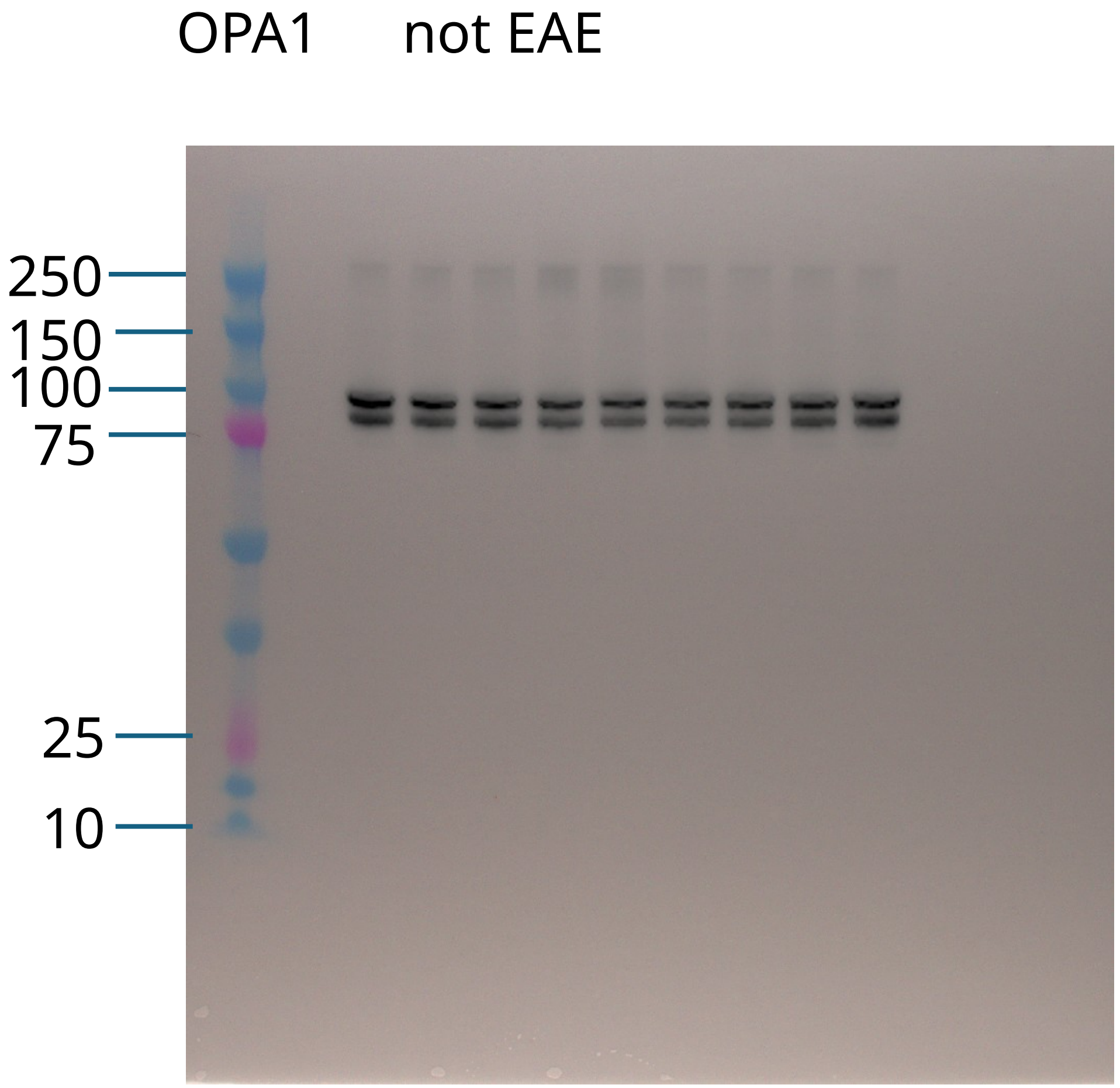


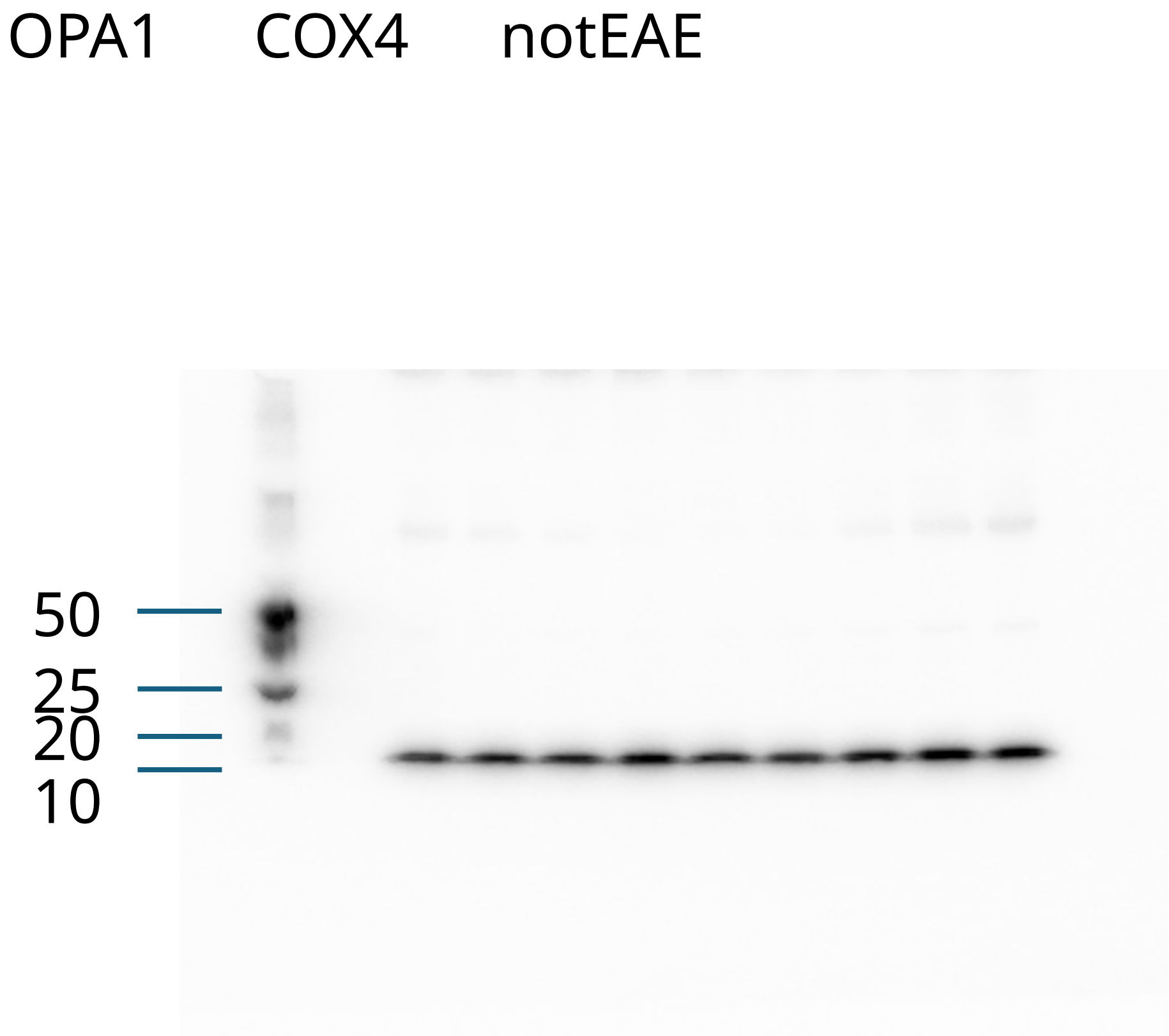

**B. MFN2**

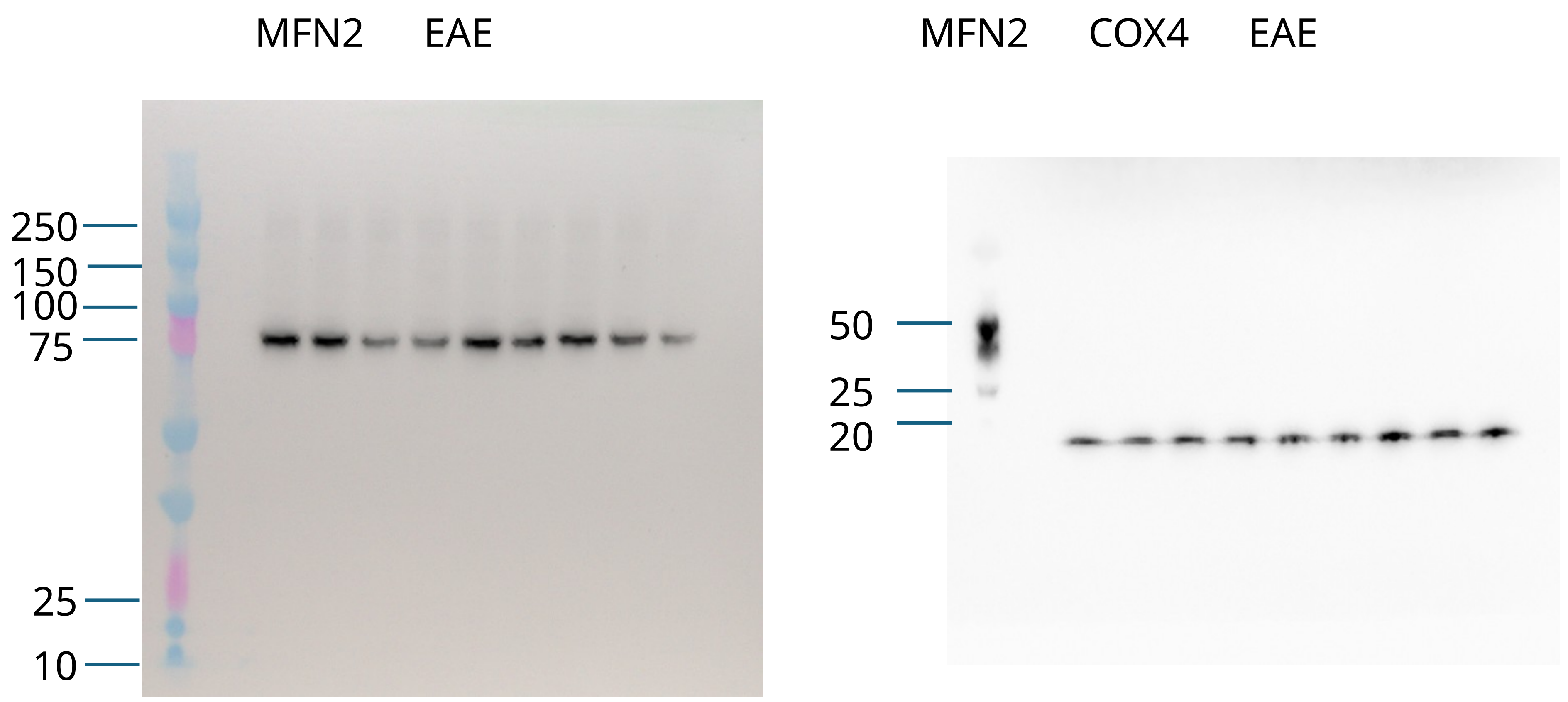

**B. MFN2**

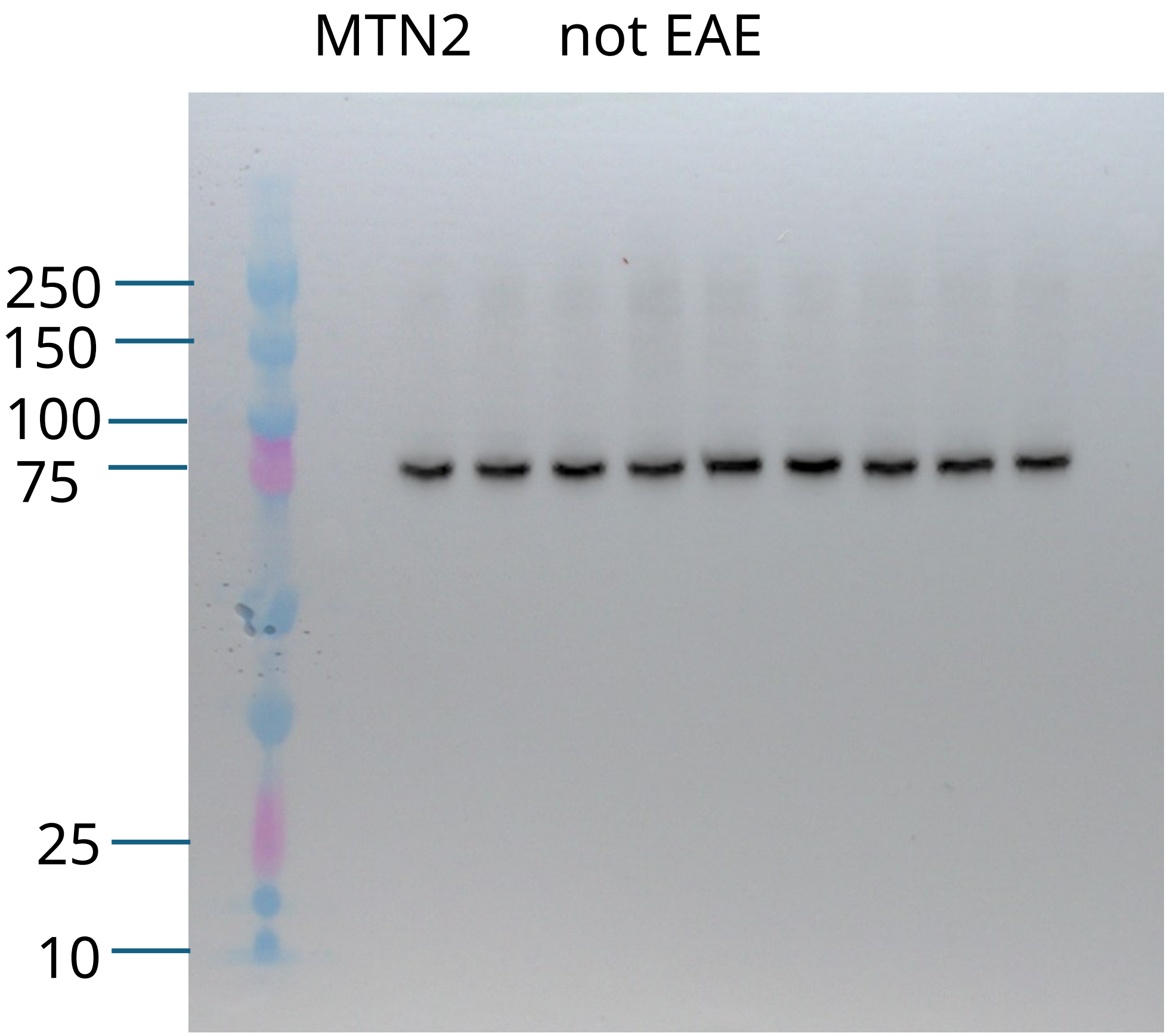


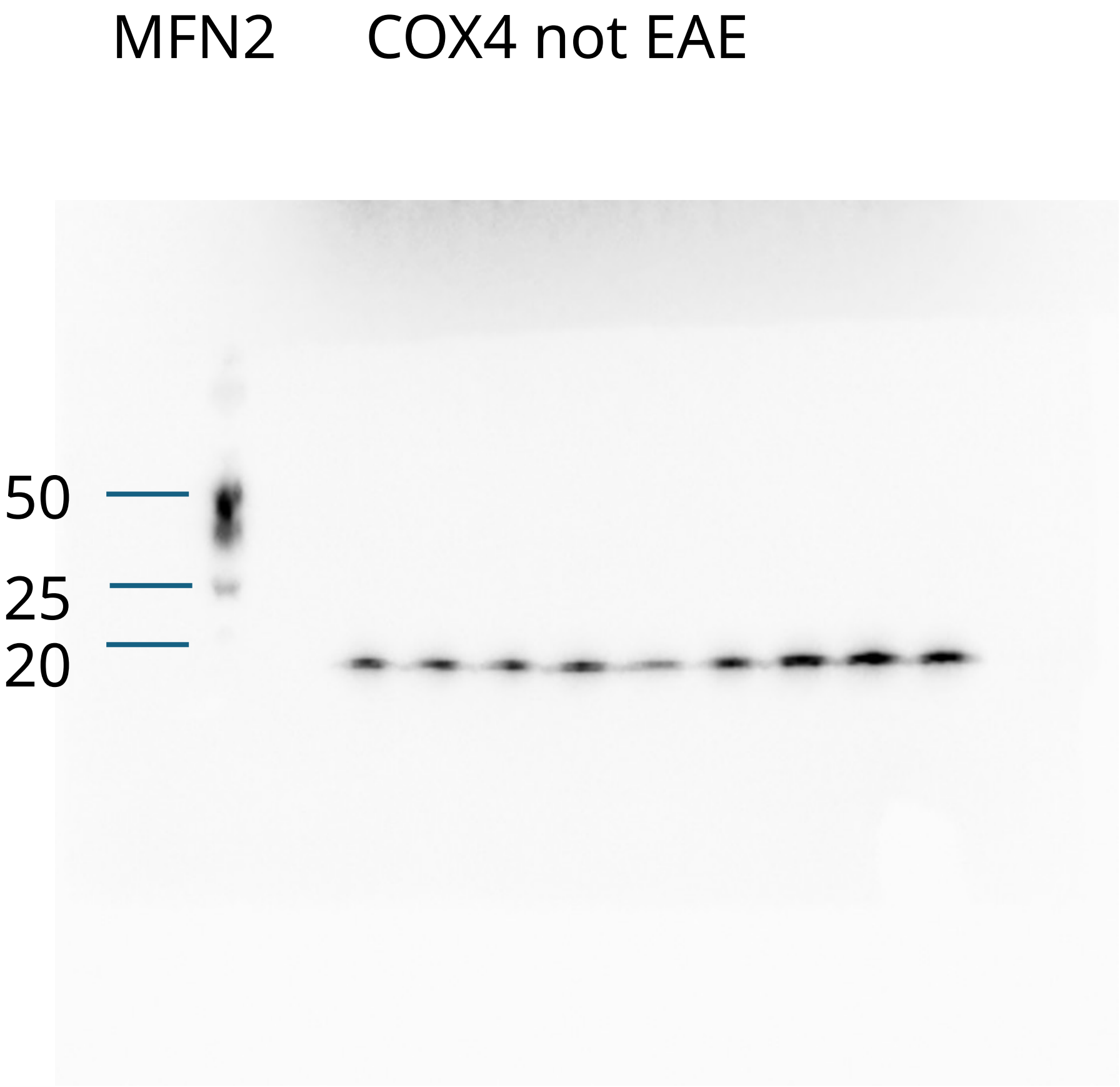

**C. DRP1**

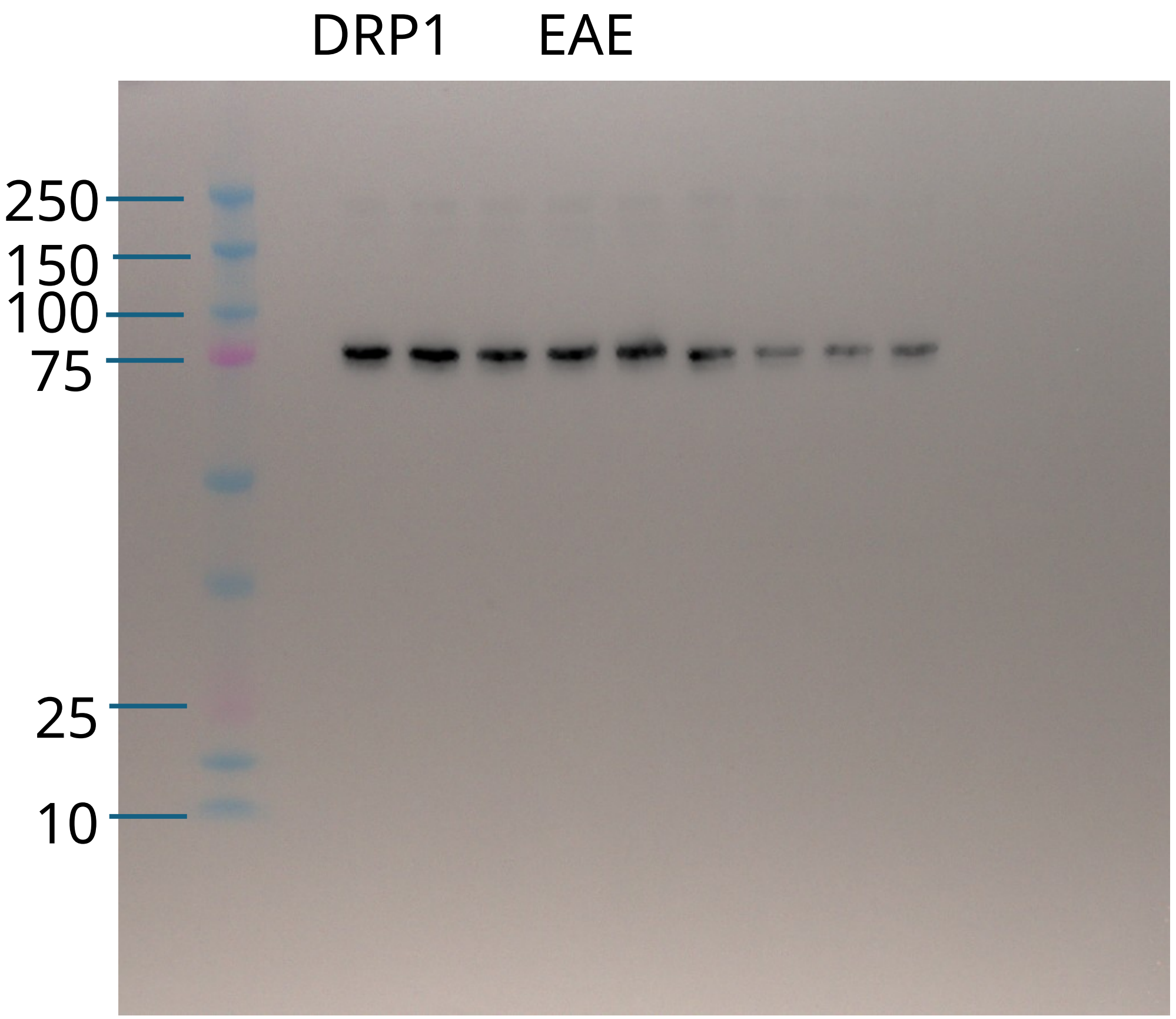


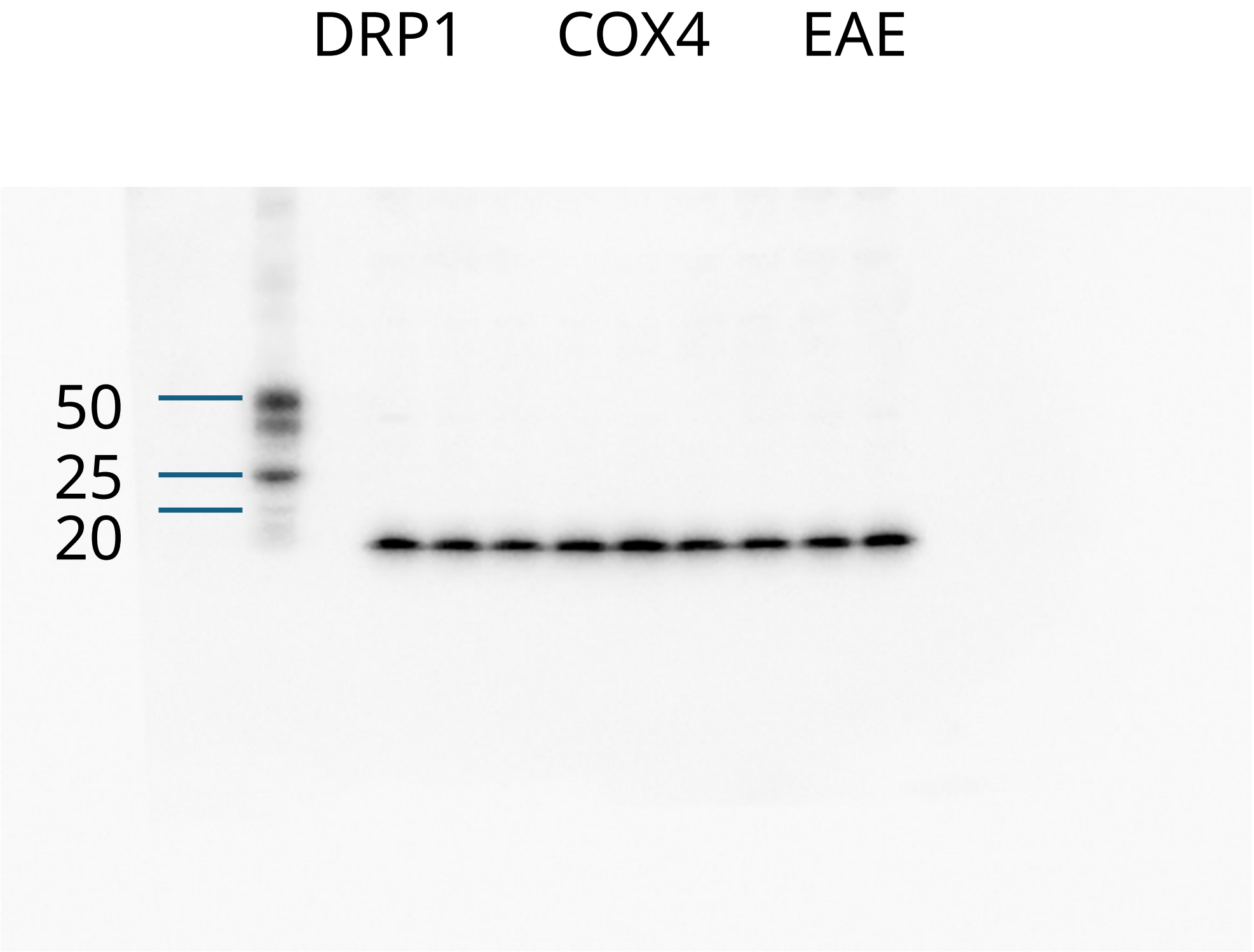

**C. DRP1**

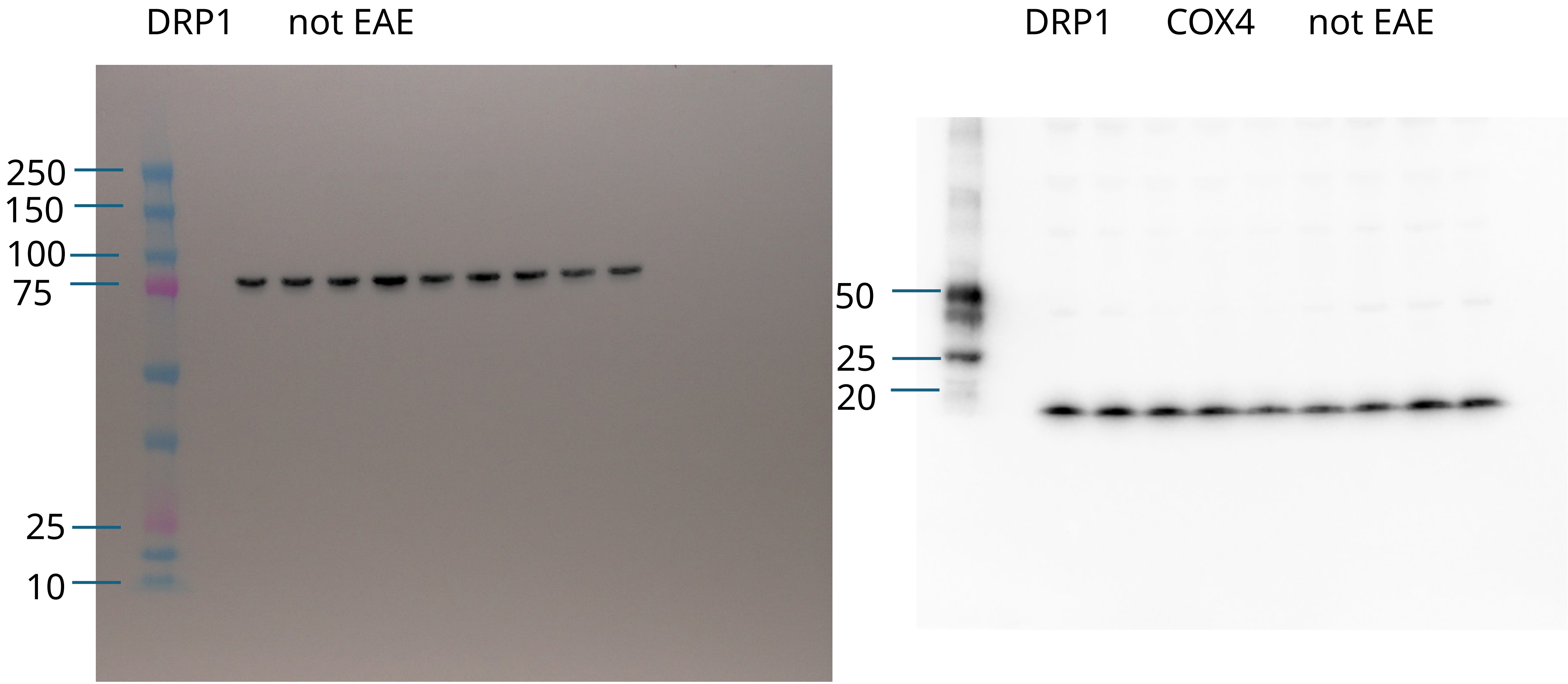

Supplement: Supplementary file 1 [file JNC-170-e70561-s001.pdf]
